# Effect of different exercise interventions on metabolic syndrome risk factors in postmenopausal women: a network meta-analysis

**DOI:** 10.3389/fphys.2025.1703881

**Published:** 2025-11-10

**Authors:** Tongyan Zhang, Anastasiia V. Kabachkova, Zifu Deng, Yishu Liang, Meng Li, Wenxue Yuan

**Affiliations:** 1 Faculty of Physical Education, Tomsk State University, Tomsk, Russia; 2 School of General Education, Dalian University of Technology, Dalian Liaoning, China

**Keywords:** postmenopausal women, exercise interventions, metabolic syndrome risk factors, body composition, network meta-analysis

## Abstract

**Objectives:**

The objective of this study was to compare and rank the effectiveness of various exercise interventions on metabolic syndrome (MetS) risk factors in postmenopausal women.

**Methods:**

A systematic search was conducted in PubMed, Cochrane, Embase, and Web of Science databases. Randomized controlled trials investigating exercise effects on MetS risk factors in postmenopausal women were included. Two reviewers screened articles, extracted data, and assessed risk of bias and strength of evidence. Analysis was performed by RStudio and Stata 16.0.

**Results:**

This study encompassed 142 RCTs with 7,967 women. The results of the network meta-analysis indicated that combined training (CT) had the greatest effect on body weight (surface under the cumulative ranking [SUCRA] = 0.897), body mass index (SUCRA = 0.923) and triglyceride levels (SUCRA = 0.783); aerobic exercise (AE) had the most significant effect on body fat percentage (SUCRA = 0.856), low-density lipoprotein cholesterol (SUCRA = 0.765), and high-density lipoprotein cholesterol levels (SUCRA = 0.814); resistance training (RT) had the greatest effect on waist circumference (SUCRA = 0.834), glucose (SUCRA = 0.929),and total cholesterol levels (SUCRA = 0.776); mind-body exercise (MBE) had the most significant effect on diastolic blood pressure (SUCRA = 0.969), systolic blood pressure (SUCRA = 0.921), and adiponectin levels (SUCRA = 0.808).

**Conclusion:**

AE, CT, RT, and MBE demonstrated varying degrees of effectiveness in improving different MetS risk factors in postmenopausal women. Selecting appropriate exercise modalities based on individual metabolic risk profiles and health goals is important to achieve optimal intervention outcomes. These findings provide valuable guidance for clinical practice. However, considering the limitations such as the low quality of evidence and high risk of bias in the included studies, the conclusions should be interpreted with caution.

**Systematic Review Registration:**

https://www.crd.york.ac.uk/PROSPERO/view/CRD42023456584, identifier CRD42023456584.

## 1 Introduction

Metabolic syndrome (MetS) is characterized by a cluster of interrelated non-communicable and chronic metabolic disorders affecting more than 1 billion individuals worldwide (Saklayen, 2018). It is widely recognized as a major contributor to cardiovascular disease, while also increasing the risk of diabetes (Watanabe and Kotani, 2020; Palmer and Toth, 2019), acute pancreatitis, venous thrombosis, and psoriatic fatty liver (Jang et al., 2009; Grandl and Wolfrum, 2018; Gisondi et al., 2018). For women, the postmenopausal period is a critical stage during which the risk of MetS increases significantly (Torréns et al., 2009). Studies have shown that postmenopausal women have a significantly higher risk of developing MetS compared with premenopausal women (Hallajzadeh et al., 2018), which is closely related to the decline in endogenous estrogen levels caused by decreased ovarian function after menopause. Estrogen plays an important role in maintaining insulin sensitivity, regulating lipid metabolism, and inhibiting visceral fat accumulation. Its reduction can lead to insulin resistance, elevated plasma triglycerides, decreased high-density lipoprotein cholesterol (HDL-C), thereby significantly increasing the risk of developing or worsening MetS in postmenopausal women. Therefore, deepening the understanding of the risk factors for MetS (Min et al., 2022) is of particular importance to prevent and alleviate this disease in postmenopausal women.

Compared with pharmacological interventions, exercise offers significant cost-effectiveness and long-term safety in improving various risk factors of MetS. Previous studies have demonstrated that regular physical activity and long-term exercise can effectively reduce body weight (BW), lower blood pressure, and improve blood lipid profiles (Grundy, 2016; Liang et al., 2021). Among different exercise modalities, aerobic exercise (AE) and resistance training (RT) are the most common forms of exercise. Studies have found that long-term AE significantly improves BW, HDL-C, low-density lipoprotein cholesterol (LDL-C), systolic blood pressure (SBP), and diastolic blood pressure (DBP) in postmenopausal women (Kobayashi et al., 2022; Pereira et al., 2023). RT interventions can reduce levels of adipokines, particularly adiponectin (ADPN), associated with abnormal vasodilation in postmenopausal women (Ward et al., 2020). In addition to traditional AE and RT, other forms of exercise have gradually gained attention. Mind-body exercise (MBE), which integrates breathing regulation, physical movement, and psychological adjustment, has gained increasing attention in recent years. Tai Chi, yoga, and qigong are currently popular MBEs (Tan L. et al., 2023; Han et al., 2023). Postmenopausal women have been shown to improve energy metabolism, BW, body fat (BF), and blood pressure through these exercises (Boushehri et al., 2022; Buttelli et al., 2021).

Although numerous randomized controlled trials (RCTs) have investigated the effects of exercise on improving various risk factors of MetS in postmenopausal women (Kobayashi et al., 2022; Pereira et al., 2023; Ward et al., 2020; Boushehri et al., 2022; Nunes et al., 2022), and recent meta-analyses have further confirmed the critical role of exercise in reducing MetS risk in this population (Bernal et al., 2025; Tan A. et al., 2023), evidence comparing the relative efficacy of different exercise interventions remains limited. Most existing studies have focused on single exercise modalities versus control groups or pairwise comparisons between two types of exercise, lacking systematic and comprehensive comparisons across multiple exercise interventions. Moreover, the most effective exercise strategies for improving specific MetS risk factors have not been clearly identified, and individualized intervention strategies tailored for postmenopausal women are still lacking. Therefore, exploring and comparing the specific impacts of different exercise interventions on metabolic risk factors in postmenopausal women is essential for informing the development of evidence-based exercise prescriptions. In response to these gaps, this study aims to systematically evaluate the comparative effects of various exercise interventions through a network meta-analysis (NMA).

## 2 Methods

### 2.1 Registration

This systematic review and NMA was reported following the Preferred Reporting Items for Systematic Reviews and Meta-Analyses (PRISMA) guidelines (Page et al., 2021; Hutton et al., 2015). The protocol has been registered on the International prospective register of systematic reviews (CRD42023456584).

### 2.2 Search strategy

Systematic searches were conducted in Embase, PubMed, Cochrane Library, and Web of Science for studies investigating the effect of exercise training on MetS risk factors in postmenopausal women up to December 2024. The references of included studies were also tracked. According to the PICOS (Participant, Intervention, Comparison, Outcome) principles, the strategy of combining “keywords” and “free terms” was adopted for searching, including menopause, metabolic syndrome, exercise, blood pressure, blood glucose, triglycerides, etc. A detailed example of the Web of Science search strategy is provided in Supplementary Table S1.

### 2.3 Selection of studies and eligibility criteria

After the searched studies were imported into Endnote X9, and duplicate records were removed, two reviewers (Z and L) independently screened the literature using the following inclusion and exclusion criteria. Inclusion criteria: (1) Study type: RCTs and human studies; (2) Study population: Postmenopausal women with no history of breast cancer, heart disease, or other serious medical conditions (defined as menopause lasting at least 1 year or follicle-stimulating hormone (FSH) level >30 IU/L) women; (3) Intervention types: AE, aimed at improving cardiorespiratory efficiency and capacity (Mersy, 1991); RT, aimed at increasing intensity, strength, endurance, and skeletal muscle size (Howley, 2001); combined training (CT), involving both AE and RT; MBE can be defined as an exercise methodology that integrates strategies for improving mental and physical health (Tan L. et al., 2023). (4) Outcome measures: BW, body mass index (BMI), BF%, waist circumference (WC), SBP, DBP, Glu, LDL-C, HDL-C, total cholesterol (TC), triglyceride (TG), ADPN and leptin; (5) Accepted articles must be published in English. Exclusion criteria: (1) Duplicates; (2) Systematic reviews, meta-analyses, and conference papers; (3) Animal studies; (4) Inaccessible full text and relevant information; Literature retrieval and screening were conducted independently by two reviewers (Z and L). To improve efficiency, an initial automated screening was performed using the search function of EndNote to exclude records identified as conference papers, systematic reviews, or meta-analyses based on title and abstract. The remaining records were then manually screened by the two reviewers according to the predefined criteria. In case of any discrepancies, a consensus was reached through discussion or consultation with a third reviewer.

### 2.4 Data extraction

The data of eligible studies was independently extracted and organized into Excel 2019 by two reviewers (Z and L), including (1) Basic information: Title, first author, year of publication, and study type; (2) Participant information: Country, average age, sample size, and health status; (3) Information on exercise interventions: Type of exercise, duration of intervention. (4) Outcome measures: BW, BF%, BMI, WC, SBP, DBP, Glu, LDL-C, HDL-C, TC, TG, ADPN, Leptin. Data extraction was performed independently by two reviewers (Z and L), and any discrepancies were resolved through discussion or consultation with a third reviewer to reach a consensus.

### 2.5 Risk of bias and strength of evidence assessment

The risk of bias in the included RCTs was assessed employing the Version 2 of the Cochrane risk-of-bias tool for randomized trials, as described in the sixth edition of the Cochrane Handbook for Systematic Reviews of Interventions published in 2019 (Sterne et al., 2019). The included studies were assessed for quality from the following aspects: (1) bias arising from the randomization process, (2) bias due to deviations from intended interventions, (3) bias due to missing outcome data, (4) bias in measurement of the outcome, and (5) bias in selection of the reported result. The risk-of-bias judgments for each domain were “low risk of bias,” “some concerns,” or “high risk of bias.” The quality assessment was independently performed by two reviewers (Z and L). In case of any discrepancies, a consensus was reached through discussion or consultation with a third reviewer.

The credibility of the comparative results was assessed using the Confidence in Network Meta-Analysis (CINeMA) tool (Nikolakopoulou et al., 2020). Evaluations were conducted across the following six domains: (1) within-study bias, (2) indirectness, (3) reporting bias, (4) imprecision, (5) inconsistency, and (6) heterogeneity. Each domain was rated as “no concerns” (no downgrade), “some concerns” (one-level downgrade), or “major concerns” (two-level downgrade) based on the severity of bias. The overall confidence rating was categorized into four levels: very low, low, moderate, and high. The quality assessment was independently performed by two reviewers (Z and D). In case of any discrepancies, a consensus was reached through discussion or consultation with a third reviewer.

### 2.6 Data synthesis and analysis

Pairwise meta-analysis was performed using RStudio software. The standardized mean difference (SMD) with 95% confidence intervals (CI) was selected as the effect measure for continuous outcomes, and a random-effects model was applied to pool effect sizes across studies. A 95% CI represented each effect size. *I* ^*2*^ was used to determine the heterogeneity of effect indicators among different studies quantitatively. An *I* ^*2*^ > 50% or *P* < 0.10 for the Q test was interpreted as indicating substantial heterogeneity (Higgins et al., 2011).

The NMA was conducted using RStudio and Stata 16.0. Mean difference (MD) and 95% CI were used as the effect sizes for outcome measures, and the same measurement units were used. The network map command in Stata 16.0 was employed to generate network diagrams, funnel plots, and cumulative probability plots. Network diagrams illustrate connections between various training interventions, with nodes representing the different interventions, and edges indicating the connections or relationships between them). Funnel plots display the relationship between sample sizes and effect sizes of studies, along with potential publication bias. The surface under the cumulative ranking curve (SUCRA) was used to illustrate the ranking probabilities of the exercise interventions for each outcome. A NMA was carried out using a random-effects model based on the Bayesian framework. This model adopted the Markov Chain Monte Carlo (MCMC) method to obtain non-informative uniform and normal prior distributions. Model parameters were set as follows: four chains were used (Hamra et al., 2013; Bois, 2013), with a step size of 1, an annealing count of 20,000, and a simulation iteration of 50,000 (Dias et al., 2012). Model fit was assessed using the Deviance Information Criterion (DIC). A dDIC value less than 10 was considered to indicate no significant global inconsistency (Veroniki et al., 2013). When the network diagram had a closed loop, local inconsistency was examined through node-splitting analysis.

## 3 Results

### 3.1 Study selection

**FIGURE 1:**
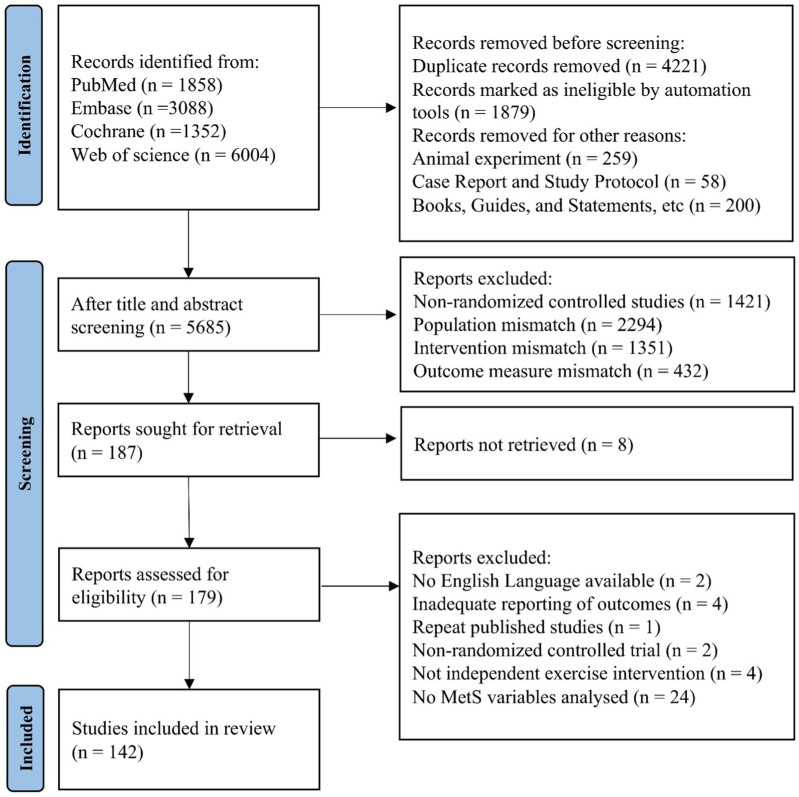
PRISMA flow diagram of the study selection process.

A total of 12,302 articles were retrieved from database searches and imported into EndNote X9. After removing 4,221 duplicate records, 5,498 articles were excluded based on title and abstract screening, due to reasons such as reviews, non-RCTs, animal studies, and non-English publications, leaving 187 articles for further full-text screening. After reading the full text, an additional 37 articles were excluded for reasons such as missing data, inadequate reporting of outcomes and not involving postmenopausal women. Finally, 142 articles were included in the review and NMA analysis. The PRISMA flowchart is presented in Figure 1.

### 3.2 Characteristics of the included studies

A total of 142 studies were included, involving 7,967 women, with 3,203 in the experimental group and 4,764 in the control group (CON). The mean age was 60.22 ± 6.38 years. These studies were conducted in various countries, including Brazil (29 studies), United States (21 studies), Japan (15 studies), Korea (15 studies), Iran (13 studies), Canada (10 studies), Poland and Spain (6 studies each), China (4 studies), Portugal, Turkey and Sweden (3 studies each), and Germany and France (2 studies each), Algeria, Australia, Chile, Egypt, Finland, Libya, Republic of South Africa, Sydney, Thailand and United Kingdom (1 study each). The experimental group used five training interventions: AE (71 studies), RT (39 studies), CT (40 studies), and MBE (18 studies), while the CON maintained their usual daily activities. The basic characteristics of the included studies are presented in Supplementary Table S2.

### 3.3 Risk of bias 2 (ROB) quality evaluation

Among the 142 included studies, 22.5% had “low risk of bias”, 39.4% had “some concerns”, and 38% had “high risk of bias”. Regarding different domains of bias, the bias arising from the randomization process was categorized as “low risk” in 29.6% of studies, “some concerns” in 65.5% of studies, and “high risk” in 4.9% of studies. Deviations from intended interventions resulted in a bias categorized as “low risk” in 72.5% of studies, “some concerns” in 7% of studies, and “high risk” in 20.4% of studies. Bias due to missing outcome data was classified as “low risk” in 97.2% of studies and “high risk” in 2.8% of studies. Bias in measurement of the outcome was identified as “low risk” in 83.8% of studies, “some concerns” in 2.1% of studies, and “high risk” in 14.1% of studies. Bias in selection of the reported results was rated as “low risk” in 98.6% of studies and “some concerns” in 1.4% of studies. The primary sources of risk were as follows: 23 studies mentioned random allocation using random number tables or computer programs, 2 study described random grouping by independent personnel, while the remaining 117 studies did not provide detailed descriptions of the randomization method. Blinding participants in exercise-related studies had some difficulties; therefore, only 18 studies reported blinding procedures, with 10 studies using double blinding, 8 studies using single blinding, and the remaining studies lacking detailed descriptions. 33 RCTs had missing outcome data, 27 studies provided evidence that the results were not influenced by missing data, 33 study reported participant withdrawal due to health, illness, death reasons, and 6 studies did not provide evidence that results were not affected by missing data. All studies adopted appropriate outcome measurement methods, avoiding selective reporting of results. Supplementary Figure S1 represents the ROB diagram. Using CINeMA, most pairwise comparisons were found to have low confidence levels, with only a few demonstrating moderate to high confidence. The CINeMA results in Supplementary Table S3.

### 3.4 Pairwise meta-analysis and NMA

**FIGURE 2:**
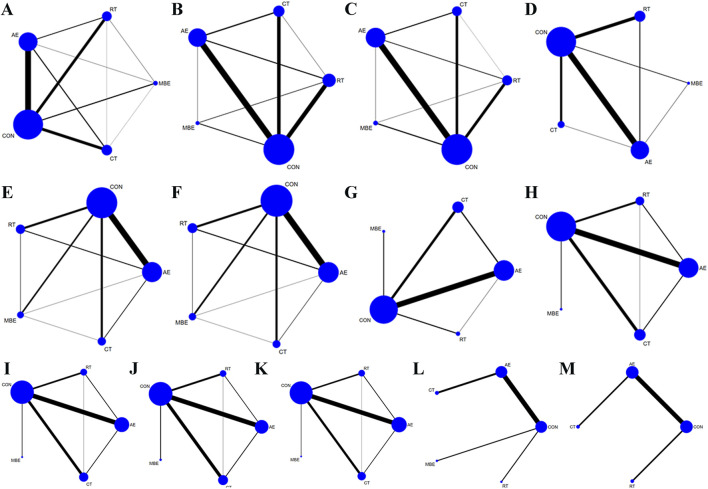
Network graph. Each node represents one Training type. The lines between the dots indicate a direct comparison between the two modes of motion, with thicker lines for more studies and thinner lines for fewer studies. **(A)** Body weight **(B)** Body fat% **(C)** Body Mass Index **(D)** Waist circumference **(E)** Diastolic blood pressure **(F)** Systolic blood pressure **(G)** Glucose **(H)** Low-density lipoprotein cholesterol **(I)** High-density lipoprotein cholesterol **(J)** Total cholesterol **(K)** Triglyceride **(L)** Adiponectin **(M)** Leptin; AE, aerobic exercise; RT, resistance training; CT, combined training; MBE, Mind-body exercise; CON, control group.

The included 142 studies discussed four different training interventions: AE, RT, CT and MBE. The network structure diagram illustrating the relationships between these interventions is presented in Figures 2A–M. In the Figure, the thickness of the lines in the diagram can reflect the number of pairwise comparisons among the interventions. Additionally, the size of the circles representing the interventions can be proportional to the number of participants included in each intervention. The difference in DIC values between the consistency model and the inconsistency model was <5, indicating the absence of local inconsistency. For outcomes with closed loops, a node-splitting analysis was conducted, which revealed that all p-values were >0.05, indicating the absence of local inconsistency.

#### 3.4.1 Body composition (BW, BF%, BMI and WC)

**FIGURE 3:**
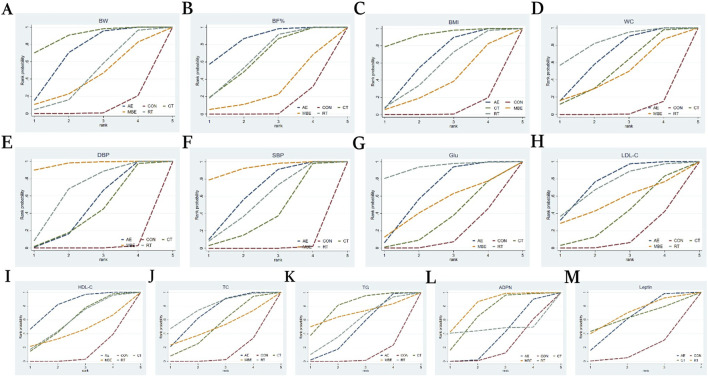
Cumulative ranking probability graph. The surface under the cumulative ranking curve (SUCRA) value is the probability each treatment has of being among the best of those in the network, with larger values representing higher ranking probabilities. **(A)** Body weight **(B)** Body fat% **(C)** Body Mass Index **(D)** Waist circumference **(E)** Diastolic blood pressure **(F)** Systolic blood pressure **(G)** Glucose **(H)** Low-density lipoprotein cholesterol **(I)** High-density lipoprotein cholesterol **(J)** Total cholesterol **(K)** Triglyceride **(L)** Adiponectin **(M)** Leptin.

BW was reported in 102 studies involving 4,755 participants and four interventions: AE, RT, CT and MBE. Pairwise analysis indicated that exercise interventions were effective for reducing BW, with an overall *I* ^*2*^ value of 42.2% (Supplementary Table S4). Compared to the CON, AE (MD = −1.43, 95% CI: −1.97, −0.9) and CT (MD = −1.84, 95% CI: −2.71, −0.95) were more effective in improving BW among postmenopausal women. No statistically significant differences were found in pairwise comparisons between other interventions (Supplementary Table S5). The ranking order of interventions in terms of improving BW was as follows: CT (SCURA = 0.897), AE (SCURA = 0.703), RT (SCURA = 0.437) (Figure 3A).

BF% was reported in 56 studies involving 2057 participants and four interventions: AE, RT, CT and MBE. The pairwise meta-analysis demonstrated that exercise interventions significantly reduced BF%, with an overall *I* ^*2*^ value of 75.8% (Supplementary Table S4). Compared to the CON, AE (MD = −2.22, 95% CI: −3.15, −1.28), CT (MD = −1.71, 95% CI: −2.96, −0.46) and RT (MD = −1.77, 95% CI: −2.85, −0.7) were more effective in improving BF% among postmenopausal women. No statistically significant differences were found in pairwise comparisons between other interventions (Supplementary Table S5). The ranking order of interventions in terms of improving BF% was as follows: AE (SCURA = 0.856), RT (SCURA = 0.656), CT (SCURA = 0.637) (Figure 3B).

BMI was reported in 96 studies involving 4,494 participants and four interventions: AE, RT, CT and MBE. Pairwise analysis indicated that exercise interventions were were associated with a significant decrease in BMI, with an overall *I* ^2^ value of 60.9% (Supplementary Table S4). Compared to the CON, AE (MD = −0.52, 95% CI: −0.78, −0.26), CT (MD = −0.83, 95% CI: −1.3, −0.35), and RT (MD = −0.44, 95% CI: −0.88, 0) were ineffective at improving BMI among postmenopausal women. No statistically significant differences were found in pairwise comparisons between other interventions (Supplementary Table S5). The ranking order of interventions in terms of improving BMI was as follows: CT (SCURRT = 0.923), AE (SCURCT = 0.628), RT (SCURMBE = 0.532) (Figure 3C).

WC was reported in 42 studies involving 2,389 participants and four interventions: AE, RT, CT and MBE. The pairwise meta-analysis demonstrated that exercise interventions effectively lowered WC, with an overall *I* ^*2*^ value of 63.4% (Supplementary Table S4). Compared to the CON, AE (MD = −2.25, 95% CI: −3.23, −1.29), CT (MD = −1.77, 95% CI: −3.56, −0.02) and RT (MD = −2.8, 95% CI: −4.36, −1.28) were more effective in improving BMI among postmenopausal women. No statistically significant differences were found in pairwise comparisons between other interventions (Supplementary Table S5). The ranking order of interventions in terms of improving WC was as follows: RT (SCURT = 0.834), AE (SCURCT = 0.659), and CT (SCURST = 0.509) (Figure 3D).

#### 3.4.2 Blood pressure (DBP, SBP)

DBP was discussed in 62 studies involving 3,000 participants and four interventions: AE, RT, CT and MBE. The pairwise meta-analysis demonstrated that exercise interventions had a significant beneficial effect on DBP. The heterogeneity, with an overall *I* ^*2*^ value of 88.0% (Supplementary Table S4). Compared to the CON, AE (MD = −2.5, 95% CI: −3.8, −1.18), CT (MD = −2.21, 95% CI: −4.41, −0.02), MBE (MD = −5.47, 95% CI: −7.99, −3.02) and RT (MD = −3.4, 95% CI: −5.55, −1.3) MBE showed a more favorable effect on DBP compared to AE (MD = −2.97, 95% CI: −5.75, −0.27). MBE outperformed CT (MD = −3.26, 95% CI: −6.51, −0.06) in improving DBP. No statistically significant differences were found in pairwise comparisons between other interventions (Supplementary Table S6). The ranking order of interventions in terms of improving DBP was as follows: MBE (SCURMBE = 0.969), RT (SCURRT = 0.661), AE (SCURAE = 0.457) (Figure 3E).

SBP was reported in 64 studies involving 2,960 participants and six interventions: AE, RT, CT and MBE. The pairwise meta-analysis demonstrated that exercise interventions had a significant beneficial effect on SBP. The heterogeneity, with an overall *I* ^*2*^ value of 90.5% (Supplementary Table S4). Compared to the CON, AE (MD = −5.11, 95% CI: −7.02, −3.17), CT (MD = −3.47, 95% CI: −6.74, −0.18), MBE (MD = −7.21, 95% CI: −10.63, −3.74) and RT (MD = −4.58, 95% CI: −7.74, −1.46) were more effective in improving SBP in postmenopausal women. No statistically significant differences were found in pairwise comparisons between other interventions (Supplementary Table S6). The ranking order of interventions in terms of improving SBP was as follows: MBE (SCURMBE = 0.921), AE (SCURAE = 0.643), RT (SCURRT = 0.544) (Figure 3F).

#### 3.4.3 Glu

Glu was reported in 37 studies involving 2,102 participants and four interventions: AE, RT, CT and MBE. The pairwise meta-analysis demonstrated that exercise interventions significantly decreased Glu, with an overall *I* ^*2*^ value of 63.3% (Supplementary Table S4). Compared to the CON, AE (MD = −3.66, 95% CI: −6.32, −1.03) and RT (MD = −8.24, 95% CI: −15.1, −1.52) showed a greater advantage in improving Glu in postmenopausal women. No statistically significant differences were found in pairwise comparisons between other interventions (Supplementary Table S7). The ranking order of interventions in terms of improving Glu was as follows: RT (SCURRT = 0.929), AE (SCURAE = 0.640), MBE (SCURHIIT = 0.485) (Figure 3G).

#### 3.4.4 Cholesterol and blood lipid (LDL-C, HDL-C, TC, and TG)

LDL-C was reported in 46 studies involving 1,960 participants and four interventions: AE, RT, CT and MBE. The pairwise meta-analysis demonstrated that exercise interventions significantly reduced LDL-C, with an overall *I* ^2^ value of 76.9% (Supplementary Table S4). Compared to the CON, AE (MD = −8.36, 95% CI: −13.11, −3.9) showed a greater advantage in improving LDL-C in postmenopausal women. No statistically significant differences were found in pairwise comparisons between other interventions (Supplementary Table S8). The ranking order of interventions in terms of improving LDL-C was as follows: AE (SCURAE = 0.765), RT (SCURRT = 0.726), MBE (SCURMBE = 0.528) (Figure 3H).

HDL-C was mentioned in 50 studies involving 2,072 participants and four interventions: AE, RT, CT and MBE. The pairwise meta-analysis demonstrated that exercise interventions significantly increased HDL-C, with an overall *I* ^2^ value of 72.4% (Supplementary Table S4). In terms of raising HDL-C levels among postmenopausal women, AE (MD = 3.23, 95% CI: 1.53, 4.92) demonstrated a stronger benefit over the CON. No statistically significant differences were observed in pairwise comparisons between other interventions (Supplementary Table S8). The ranking order of interventions in terms of improving HDL-C was as follows: AE (SCURAE = 0.814), RT (SCURT = 0.579), CT (SCURCT = 0.578) (Figure 3I).

TC was discussed in 52 studies involving 2, 093 participants and five interventions: AE, RT, CT and MBE. The pairwise meta-analysis demonstrated that exercise interventions significantly reduced TC, with an overall *I* ^*2*^ value of 66.6% (Supplementary Table S4). In terms of improving TC levels among postmenopausal women, AE (MD = −6.26, 95% CI: −10.56, −2.18) and RT (MD = −7.69, 95% CI: −15.32, −0.5) outperformed the CON. No statistically significant differences were observed in pairwise comparisons between other interventions (Supplementary Table S8). The ranking order of interventions in terms of improving TC was as follows: RT (SCURRT = 0.776), AE SCURAE = 0.684), CT (SCURST = 0.475) (Figure 3J).

TG was analyzed in 59 studies involving 2,623 participants and four interventions: AE, RT, CT and MBE. The pairwise meta-analysis demonstrated that exercise interventions markedly decreased TG, with an overall *I* ^*2*^ value of 76.8% (Supplementary Table S4). In terms of raising TG levels among postmenopausal women, AE (MD = −5.62, 95% CI: −10.68, −0.54) and CT (MD = −11.49, 95% CI: −19.53, −3.65) showed a greater advantage over the CON. No statistically significant differences were observed in pairwise comparisons between other interventions (Supplementary Table S8). The ranking order of interventions in terms of improving TG was as follows: CT (SCURCT = 0.783), MBE (SCURMBE = 0.686), RT (SCURAE = 0.520) (Figure 3K).

#### 3.4.5 ADPN and leptin

ADPN was discussed in 9 studies involving 1,077 participants and four interventions: AE, RT, CT and MBE. The pairwise meta-analysis demonstrated that exercise interventions did not significantly elevated ADNP, with an overall *I* ^*2*^ value of 63.1% (Supplementary Table S4). Among postmenopausal women, MBE (MD = 1.84, 95% CI: 0.32, 3.31) exhibited a more notable improvement in ADPN levels than AE. Similarly, MBE (MD = 1.96, 95% CI: 0.58, 3.37) showed a stronger advantage over the CON in improving ADPN among postmenopausal women. Pairwise comparisons of other interventions did not reveal any statistically significant changes (Supplementary Table S9). The ranking order of interventions in terms of improving ADPN was as follows: MBE (SCURMBE = 0.808), CT (SCURCT = 0.677), RT (SCURRT = 0.511) (Figure 3L).

Ten studies reported Leptin, involving 906 participants and three interventions: AE, RT, and CT. The pairwise meta-analysis demonstrated that exercise interventions caused a significant decrease in leptin, with an overall *I* ^*2*^ value of 83.9% (Supplementary Table S4). No statistically significant differences were found in pairwise comparisons between the interventions (Supplementary Table S9). The ranking order of interventions in terms of improving Leptin was as follows: RT (SCURRT = 0.671), CT (SCURCT = 0.626), AE (SCURAE = 0.582) (Figure 3M).

### 3.5 Publication bias or small sample effect test

**FIGURE 4:**
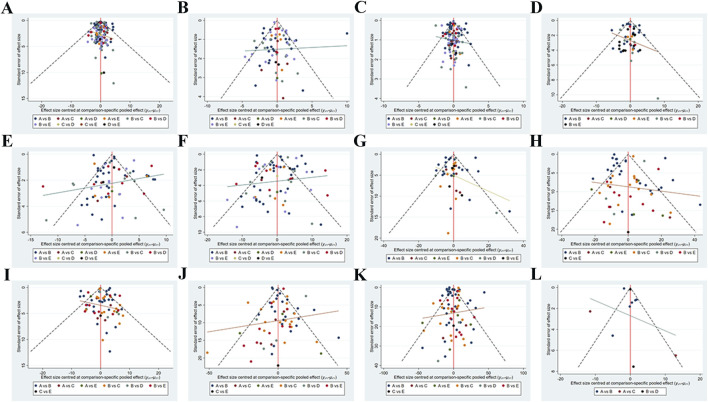
Network meta-analysis funnel graph. Publication Bias or Small Sample Effect Test. **(A)** Body weight **(B)** Body fat% **(C)** Body Mass Index **(D)** Waist circumference **(E)** Diastolic blood pressure **(F)** Systolic blood pressure **(G)** Glucose **(H)** Low-density lipoprotein cholesterol **(I)** High-density lipoprotein cholesterol **(J)** Total cholesterol **(K)** Triglyceride **(L)** Leptin.

Funnel plots were used to examine publication bias for the indicators involved in this study (Figures 4A–L). No test was conducted for ADPN studies due to a sample size of less than 10. The funnel plots for BW, BF%, BMI, WC, DBP, SBP, Glu, LDL-C, HDL-C, TC, TG and Leptin indicators were basically symmetrical.

## 4 Discussion

In this NMA, a total of 142 studies with 7,967 participants on studies that implemented exercise training interventions for postmenopausal women with at least one MetS risk factor, in order to evaluate the effectiveness of different training modalities in improving these risk factors. We have several findings. Compared to the CON, AE showed greater advantages in improving BW, BF%, BMI, WC, DBP, SBP, Glu, LDL-C, HDL-C, TC and TG in postmenopausal women. CT were more effective than CON in improving BW, BF%, BMI, WC, DBP, SBP and TG in postmenopausal women. RT exhibited better effects CON in improving BF%, BMI, WC, SBP, Glu and TC. For reducing BW, BMI and TG, CT was most likely to be the most effective training. For reducing BF% and improving LDL-C and HDL-C, AE was most likely to be the most effective training. In terms of improving WC, Glu and TC, RT ranked first. Lastly, for DBP, SBP and ADPN improvement, MBE demonstrated the greatest superiority. It is evident that different training modalities have a significant effect on MetS risk factors in postmenopausal women. However, the effectiveness of these modalities in improving each specific risk factor varies. The optimal exercise interventions for MetS risk factors in postmenopausal women in Supplementary Figure S2.

Regarding the improvement of body composition in postmenopausal women, both AE and CT are beneficial, while RT can specifically improve BF% and WC. Previous studies have confirmed that AE is an effective way to reduce weight and improve BF%, while RE is an effective way to reduce WC (Schroeder et al., 2019). For example, Nordic walking, a form of exercise that combines walking with cross-country skiing using poles to propel oneself across the ground, has been found to reduce weight and BMI gain in postmenopausal women (Hagner-Derengowska et al., 2015; Mathieson and Lin, 2014). Compared to isolated Pilates or dietary interventions, Nordic walking is more effective in reducing weight (6.4%), blood glucose (3.8%), and lipoprotein (10.4%–16.7%) during menopause (Hagner-Derengowska et al., 2015). A recent meta-analysis on AE, RT, and CT found that both AE and RT can reduce fat content (Blumenthal et al., 2000). Orsatti et al. observed a reduction of approximately 10% in BF % after a 16-week RT in sedentary women (Carneiro et al., 2021). These findings are consistent with the results of our study and support the adoption of AE, RT and CT to better improve body composition in postmenopausal women. According to the network meta-analysis, AE is optimal for improving BF%, possibly because it promotes fat oxidation through sustained energy expenditure and enhanced lipid metabolism (Chen et al., 2025). RT preferentially improves WC, which could be related to strengthening core muscles and increasing local metabolism, thereby facilitating the reduction of abdominal fat. CT is the most effective for reducing BW and BMI due to the combined effects of multiple training modalities., combining high energy expenditure and fat oxidation with muscle maintenance or growth and an elevated basal metabolic rate, which may make it more effective than either training modality alone. These comparative effects align with their underlying physiological mechanisms. Overall, the main mechanism behind these improvements is increased energy expenditure and induction of fat breakdown through physical exercise, leading to a reduction in fat mass (Pedersen and Saltin, 2015).

Regarding the improvement of blood pressure in postmenopausal women with different training interventions, our study findings indicate that AE, CT, and MBE have significant effects on reducing DBP and SBP. MBE was identified as the most effective in improving blood pressure in postmenopausal women. A meta-analysis examining the effect of exercise on blood pressure in healthy adults found that decreases in DBP were observed after endurance training, dynamic RT, isometric RT, and CT (Cornelissen and Smart, 2013). Furthermore, another study discovered that isometric RT resulted in greater reductions in SBP, DBP, and mean arterial pressure compared to previous reports on dynamic AE or RT (Carlson et al., 2014). CT, as a combination of AE and RT, maximizes the benefits of both RT and AE. Current guidelines suggest that both treated and untreated hypertensive patients can benefit from physical fitness training, which should involve endurance training, dynamic ST, or isometric training (Pedersen and Saltin, 2015). This study found that MBE was the most effective intervention for reducing both diastolic and systolic blood pressure. The high ranking of MBE may be related to its effects on autonomic regulation, endothelial function, and the reduction of sympathetic nervous system activation. Unlike AE and RT, MBE involves controlled breathing and relaxation, which may produce stronger neurohormonal effects that contribute to blood pressure reduction. According to research, a 12-month Tai Chi intervention reduced SBP more successfully than AE (Li et al., 2024). Similarly, the Tai Chi intervention group and the non-exercise group showed a significant difference in DBP, according to another study (Huang et al., 2022). These findings suggest that MBE interventions may be beneficial for the prevention and management of elevated blood pressure in individuals with MetS. Multiple factors are involved in the blood pressure-lowering effects of exercise interventions. The first is neurohormonal, vascular and structural adaptation. The second is an antihypertensive effect by reducing sympathetic-induced vasoconstriction (Esler et al., 2001) and lowering catecholamine levels under good conditions. Thirdly, physical exercise reduces blood pressure by increasing insulin sensitivity in trained muscles and thereby alleviating hyperinsulinemia. Additionally, exercise training shares similar mechanisms for improving Glu level.

This study reveals that RT is the optimal choice for improving Glu levels in postmenopausal women, consistent with previous findings. Won et al. observed significant improvement in blood glucose levels after 12 weeks of RT (Son and Park, 2021). This is attributed to the increased insulin sensitivity in trained muscles and enhanced glucose uptake induced by muscle contractions. Holten’s research demonstrated that insulin action in skeletal muscles, both in individuals with type 2 diabetes and healthy controls, was enhanced after 6 weeks of RT, primarily due to increased content of glucose transporter-4 (GLUT4) and enhanced expression or activity of various insulin proteins (Tayebi et al., 2019; Holten et al., 2004). The mechanisms underlying the effects of exercise on insulin sensitivity and blood glucose control mainly involve increased expression of insulin receptors, improved efficiency of insulin signaling (Dela et al., 1993), elevated mRNA expression of GLUT4, enhanced glucose synthesis activity (Holten et al., 2004), increased activity of hexokinase (Coggan et al., 1993), reduced release and increased clearance rate of free fatty acids, as well as expansion of muscle capillary network and blood flow, which augments glucose transport to the muscles (Saltin et al., 1977). These factors work together to enhance the control of blood glucose.

Research on the impact of different types of training on cholesterol and blood lipids in postmenopausal women suggested that AE has a notable positive effect on blood lipid levels in this population, and is associated with decreased LDL-C and TG levels as well as increased HDL-C levels. Additionally, both RT and AE effectively improve TC levels, with CT potentially being the most effective method for improving TG levels. Similar conclusions have been drawn in related literature. For example, Leon and Sanchez conducted a meta-analysis of 51 AE interventions lasting 12 weeks or longer, showing a 4.6% increase in HDL-C levels, a 3.7% decrease in TG levels, and a 5% decrease in LDL-C levels (Leon and Sanchez, 2001). When premenopausal women conducted 14 weeks of RT, Prabhakaran et al. observed significant reductions in TC and LDL-C levels (Prabhakaran et al., 1999). Furthermore, a 12-week program of AE and RT significantly decreased TG levels, indicating that exercise has a positive impact on microcirculation. Regular physical activity has been shown to increase lipoprotein lipase activity and HDL-C levels, possibly offsetting an increase in LDL-C and TG levels (Mann et al., 2014). The results of this study suggest that AE may be the most effective intervention for improving LDL-C and HDL-C, possibly because it enhances lipid metabolism and promotes fat oxidation. Aerobic exercise can increase lipoprotein lipase activity and the expression of cholesterol transport proteins, facilitating LDL-C clearance and HDL-C production (Chen et al., 2025). In addition, AE may improve body composition and insulin sensitivity, further contributing to favorable changes in blood lipid profiles. Furthermore, CT appears to be the most effective method for reducing TG levels, likely due to the combined benefits of multiple exercise modalities. Compared with either training modality alone, the synergistic effects of increased energy expenditure, enhanced fat oxidation, greater muscle mass, and improved insulin sensitivity may collectively contribute to greater reductions in plasma TG.

As one measure of plasma adipokine levels, ADPN levels in postmenopausal women appear to be significantly positively impacted by MBE, according to this study. Several studies have demonstrated that yoga intervention can improve ADPN levels, reduce serum lipids levels, and control risk factors for MetS in obese postmenopausal women (Supriya et al., 2018; Lee et al., 2012). Chobanian et al. observed favorable modulation of adipokines in MetS participants with elevated blood pressure after yoga intervention (Chobanian et al., 2003). These results indicated that MBE may exert protective effects in women with metabolic syndrome by improving inflammatory status and the secretion profile of adipokines. This also suggests that MBE may not only have advantages in blood pressure regulation but could also broadly influence metabolic risk through endocrine pathways. While numerous studies have reported on the role of adipokines, further research is needed to delve into their signaling pathways to uncover how they interact with each other.

This study holds significant implications for both future clinical practice and scientific research. The different types of exercise have different advantages in improving various risk factors of MetS among postmenopausal women. Therefore, in clinical practice, developing personalized exercise programs based on patients’ specific metabolic profiles and health goals represents a more targeted and efficient risk intervention strategy. For individuals whose primary objectives are weight and lipid metabolism control, CT is recommended. AE is more suitable for improving body composition and regulating cholesterol levels. RT demonstrates outstanding effects in improving central obesity and glucose metabolism, while MBE is more effective for blood pressure management and enhancing overall cardiometabolic health. Integrating multiple forms of exercise may comprehensively promote metabolic health in postmenopausal women. Future research should further explore the effects of different exercise combinations, intervention durations, and exercise dosages on MetS and cardiovascular diseases. Moreover, more rigorous experimental studies are needed to establish precise exercise intervention guidelines for postmenopausal women with MetS.

## 5 Limitations

This study has several limitations. First, the nature and shortcomings of the included evidence constitute a major limitation. Although all studies were eligible RCTs and the risk of bias was systematically assessed, some studies reported insufficient details on randomization and blinding, resulting in a high risk of bias that may have led to overestimation of effect sizes. Moreover, most results were based on low to very low certainty of evidence, primarily reflecting heterogeneity and inconsistency among studies, and therefore the conclusions should be interpreted with caution. Second, only English-language studies were included. Although translation tools were considered, non-English studies were excluded due to potential terminology and semantic inaccuracies, which may have introduced language bias and affected the comprehensiveness and representativeness of the findings. In addition, this study did not consider key exercise characteristics such as intensity, frequency, and duration, nor did it account for factors such as body composition, dietary patterns, and quality of life, which are closely related to determining the optimal exercise interventions for postmenopausal women; these aspects warrant further evaluation in future research. Last, so publication bias assessment was not conducted due to the limited number of included studies on ADPN.

## 6 Conclusion

Exercise training can effectively improve key MetS risk factors in postmenopausal women. Among the exercise modalities analyzed, CT appears most effective for reducing BW, BMI and TG levels, AE is preferable for improving BF% and lipid profile, MBE is more effective in lowering blood pressure and increasing ADPN levels, and RT shows benefits in reducing WC, Glu, and TC. These findings can guide healthcare professionals in designing personalized exercise prescriptions for postmenopausal women, thereby enhancing the clinical application of exercise interventions. However, considering the limitations such as the low quality of evidence and high risk of bias in the included studies, and the complexity of this population and exercise prescription (e.g., duration, intensity), the conclusions should be interpreted with caution.

## Data Availability

The original contributions presented in the study are included in the article/Supplementary Material, further inquiries can be directed to the corresponding authors.
