# Supplementary material for: Effect of different exercise interventions on metabolic syndrome risk factors in postmenopausal women: a network meta-analysis
Source: Front Physiol. 2025 Nov 10;16:1703881. doi: 10.3389/fphys.2025.1703881 (PMC12640842; doi:10.3389/fphys.2025.1703881)
Supplement: Supplementary file 1 [file Table1.docx]

Supplementary Material

# Table S1 Web of Science search strategy.

| Searches | Date Run | Results |
| --- | --- | --- |
| 1: TS=(Exercise OR Physical Activity OR Activities, Physical OR Activity, Physical OR Physical Activities OR biometric exercise OR effort OR exertion OR fitness training OR fitness workout OR physical conditioning, human OR physical exertion OR physical workout OR aerobic exercise OR aerobic dance OR aerobic dancing OR aerobics OR dancing, aerobic OR low impact aerobics OR step aerobics OR Resistance Training OR Strength Training OR Training, Strength OR Strengthening Program, Weight-Lifting OR Weight Lifting Strengthening Program OR Exercise Program, Weight-Lifting OR Weight Lifting Exercise Program OR Strengthening Program, Weight-Bearing OR Weight Bearing Strengthening Program OR Exercise Program, Weight-Bearing OR Weight Bearing Exercise Program OR resistance-type training OR strength-type exercise OR strength-type training OR High-Intensity Interval Training OR High Intensity Interval Training OR Interval Training, High-Intensity OR Training, High-Intensity Interval OR Sprint Interval Training OR Water Tai Chi Therapy OR high intensity interval training OR high-intensity intermittent training OR HIIT OR intermittent high-intensity training OR interval high-intensity training OR Water Sports OR Sport, Water OR Water Sport OR Wave Surfing OR Surfing, Wave OR Rowing OR Water Polo OR Polo, Water OR Kayaking OR Canoeing OR Boating OR Surfboarding OR Water Skiing OR Skiing, Water OR water sport OR aquatic sport OR marine sport OR sports, aquatic OR water-based sports OR watersport OR Sports OR Sport OR Athletic OR Sport OR competitive sport ) and Preprint Citation Index (Exclude – Database) | Sun Dec 15 2024 13:58:59 GMT+0800 | 4258680 |
| 2: TS=(Metabolic Syndrome OR Syndrome, Metabolic OR Insulin Resistance Syndrome X OR Syndrome X, Metabolic OR Syndrome X, Insulin Resistance OR Metabolic X Syndrome OR Syndrome, Metabolic X OR X Syndrome, Metabolic OR Dysmetabolic Syndrome X OR Syndrome X, Dysmetabolic OR Reaven Syndrome X OR Syndrome X, Reaven OR Cardiovascular Syndrome, Metabolic OR Syndrome, Metabolic Cardiovascular OR Cardiometabolic Syndrome OR Syndrome, Cardiometabolic OR metabolic syndrome X OR insulin resistance syndrome OR Blood Pressure OR Pressure, Blood OR Diastolic Pressure OR Pressure, Diastolic OR Pulse Pressure OR Pressure, Pulse OR Systolic Pressure OR Pressure, Systolic OR blood tension OR intravascular pressure OR normotension OR vascular pressure OR Lipids OR Lipid OR Waist Circumference OR Circumference, Waist OR waist size OR Body Weight OR Weight, Body OR Blood Glucose OR Blood Sugar OR Sugar, Blood OR Glucose, Blood OR glucose blood level OR glucosaemia OR glucose, blood OR glucosemia OR glycaemia OR glycemia OR normoglycaemia OR normoglycemia OR plasma glucose OR postprandial glycaemia OR postprandial glycemia OR serum glucose OR serum sugar OR Blood Vessels OR Blood Vessel OR Vessel, Blood OR blood vessel OR ramus vascularis OR vascular branch OR vascular bundle OR vessel (artery) OR vessel (blood) OR vessel (vein)) and Preprint Citation Index (Exclude – Database) | Sun Dec 15 2024 13:59:18 GMT+0800 | 6353114 |
| 3: TS=(Postmenopause OR Postmenopausal Period OR Period, Postmenopausal OR Post Menopause OR postmenopausal female OR postmenopausal women ) and Preprint Citation Index (Exclude – Database) | Sun Dec 15 2024 13:59:30 GMT+0800 | 133617 |
| 4: #3 AND #2 AND #1 and Preprint Citation Index (Exclude – Database) | Sun Dec 15 2024 13:59:40 GMT+0800 | 6004 |

# Table S2 Basic characteristics of the included studies.

| **Study** | **Country** | **Exercise Intervention** | | | | | **Number of cases** | **Age** | **Metabolic Syndrome Risk Factors** |
| --- | --- | --- | --- | --- | --- | --- | --- | --- | --- |
|  |  | **Methods** | **Duration** | **Frequency** | **Time** | **Intensity** |  |  |  |
| Abbenhardt2013 [1] | America | AE | 12Months | 5days | 45min | 70%-85% HRmax | 117 | 58.10 ± 5.00 | ADPN, Leptin |
|  |  | CON |  |  | N/A | N/A | 87 | 57.40 ± 4.40 |  |
| Agil2010 [2] | Turkey | AE | 8Weeks | 3days | 40-45min | N/A | 18 | 52.40 ± 4.70 | TG, TC |
|  |  | RT |  |  | 40-45min | N/A | 18 | 52.60 ± 3.50 |  |
| Akazawa2012 [3] | Japan | AE | 8Weeks | 3days | 40-60min | 70%-75% HRmax | 10 | 60.00 ± 2.00 | BW, TC, HDL-C, LDL-C, TG, Glu, SBP, DBP |
|  |  | CON |  |  | N/A | N/A | 10 | 61.00 ± 2.00 |  |
| Akazawa2012 [4] | Japan | AE | 8Weeks | 3days | 40-60min | 70%-75% HRmax | 11 | 59.00 ± 5.00 | BW, BMI, TC, HDL-C, LDL-C, TG, Glu, SBP, DBP |
|  |  | CON |  |  | N/A | N/A | 10 | 64.00 ± 6.00 |  |
| Akazawa2013 [5] | Japan | AE | 8Weeks | 3days | 40-60min | 70%-75% HRmax | 13 | 59.00 ±1.00 | BW, BMI, HDL-C, LDL-C, TG, SBP, DBP |
|  |  | CON |  |  | N/A | N/A | 12 | 58.00 ± 1.00 |  |
| Aragao2014 [6] | Portugal | CT | 12Meeks | 2days | 60min | AE:50%-84% HRmax  RT:70%-80% 1RM | 88 | 55.44 ± 5.11 | BW, BMI |
|  |  | CON |  |  | N/A | N/A | 70 | 56.90 ± 4.96 |  |
| Arca2014 [7] | Brazil | AE1 | 12Weeks | 3days | 50min | 50%-60% HRmax | 19 | N/A | BW, BMI, WC, TG |
|  |  | AE2 |  |  | 50min | 50%-60% HRmax | 19 | N/A |  |
|  |  | CON |  |  | N/A | N/A | 14 | N/A |  |
| Azadpour,2017 [8] | Iran. | AE | 10Weeks | 3days | 25--40min | 50%-70% HRmax | 12 | 57.58 ± 4.29 | BW, BMI, BF%, WC, SBP, DBP |
|  |  | CON |  |  | N/A | N/A | 12 | 56.58 ± 4.17 |  |
| Babiloni2024 [9] | Spain | RT | 32Weeks | 2days | 55-60min | N/A | 35 | 69.17 ± 5.71 | SDP, DBP |
|  |  | CON |  |  | 55-60min | N/A | 11 | 67.90 ± 8.60 |  |
| Baisi2017 [10] | Brazil | CT | 20Weeks | 3days | 75min | AE: 50%-60% VO2max  RT: N/A | 35 | 61.30 ± 6.40 | BW, BMI, WC, BF%, TC, TG, HDL-C, LDL-C |
|  |  | CON |  |  | N/A | N/A | 35 | 59.80 ± 7.10 |  |
| Ballesta2020 [11] | Spain | AE1 | 18Weeks | 2days | 60min | N/A | 17 | 66.30 ± 5.44 | SBP, DBP, BMI |
|  |  | AE2 |  |  | 60min | N/A | 12 | 70.00 ± 8.76 |  |
|  |  | CON |  |  | N/A | N/A | 12 | 67.40 ± 5.71 |  |
| Barbosa2019 [12] | Brazil | CT | 18Weeks | 3days | N/A | Borg: 13-14 | 19 | 60.00 ± 4.50 | SBP, DBP |
|  |  | CON |  |  | N/A | N/A | 20 | 58.45 ± 4.80 |  |
| Bello2014 [13] | Brazil | CT | 32Weeks | 3days | N/A | AE: Borg: 12–15  RT: Borg: 12–15 | 7 | 61.30 ± 6.00 | BF% |
|  |  | CON |  |  | N/A | N/A | 7 |  |  |
| Bergstrom2009 [14] | Sweden | AE | 12Months | 4-5days | 60min | Training intensity 3-4 | 113 | 58.70 ± 4.90 | BMI, WC, TG, LDL-C, HDL-C |
|  |  | CON |  |  | N/A | N/A | 117 | 58.60 ± 5.50 |  |
| Binder1996 [15] | America | CT | 11Months | 3days | >30min | AE: 60%-70% VO2max  RT: N/A | 23 | 65.00 ± 4.00 | BF%, WC, SBP, DBP, TC, HDL-C, LDL-C, TG |
|  |  | CON |  |  | N/A | N/A | 17 | 67.00 ± 4.00 |  |
| Blain2017 [16] | France | AE | 6Months | 3days | 50min | 60%-80% HRmax | 51 | 65.58 ± 4.44 | BW, BMI, BF% |
|  |  | CON |  |  | N/A | N/A | 47 | 65.78 ± 4.15 |  |
| Bocalini2009 [17] | Brazil | RT | 24Weeks | 3days | 60min | 50%-80% 1RM | 23 | 69.00 ± 9.00 | BW, BMI, BF% |
|  |  | CON |  |  | N/A | N/A | 12 | 67.00 ± 8.00 |  |
| Bouchard2009 [18] | Canada | RT | 12Weeks | 3days | N/A | N/A | 11 | 62.80 ± 3.70 | BW, BMI |
|  |  | CON |  |  | N/A | N/A | 12 | 64.40 ±4.50 |  |
| Boushehri2022 [19] | Iran | MBE | 8Weeks | 3days | 60-70min | N/A | 15 | 55.93 ± 3.76 | BMI, BF% |
|  |  | CON |  |  | N/A | N/A | 15 | 57.46 ± 4.54 |  |
| Boutcher2019 [20] | Sydney | AE | 8Weeks | 3days | 30min | 80%-85% HRmax | 20 | 54.10 ± 3.60 | BW, WC, BMI, BF%, SBP, DBP, TC, Glu |
|  |  | CON |  |  | N/A | N/A | 20 | 53.30 ± 3.40 |  |
| Buonani2019 [21] | Brazil | CT | 16Weeks | 3days | 80min | AE: N/A  RT: 70%-80% 1RM | 9 | 59.70 ± 6.00 | TG, TC, HDL-C, LDL-C |
|  |  | CON |  |  | N/A | N/A | 13 | 63.30 ± 6.60 |  |
| Carrasco_Poyatos2012 [22] | Spain | AE | 12Months | 2days | 45min | Borg: 10-15 | 29 | 58.80 ± 6.50 | BMI |
|  |  | CT |  |  | 45min | Borg: 10-15 | 34 | 55.40 ± 6.50 |  |
|  |  | CON |  |  | N/A | N/A | 30 | 56.60 ± 6.40 |  |
| Casey2007 [23] | America | RT | 18Weeks | 2days | 35min | 50% 1-RM | 13 | 58.70 ± 4.50 | SBP, DBP |
|  |  | AE |  |  | 30-40min | 65%-80% HRmax | 10 | 59.70 ± 6.50 |  |
| Chilibeck2013 [24] | Canada | CT | 24Months | 4days | N/A | AE:70% HRmax  RT: 80% 1RM | 86 | 55.30 ± 6.30 | TC, TG, Glu, DBP, SBP |
|  |  | CON |  |  | N/A | N/A | 88 | 56.40 ± 7.10 |  |
| Church2007 [25] | America | AE1 | 6Months | 3-4days | N/A | 50% HRmax | 155 | 57.70 ± 6.60 | SBP, DBP |
|  |  | AE2 |  |  | N/A | 50% HRmax | 104 | 57.30 ± 6.60 |  |
|  |  | AE3 |  |  | N/A | 50% HRmax | 103 | 56.60 ± 6.60 |  |
|  |  | CON |  |  | N/A | N/A | 102 | 57.20 ± 5.80 |  |
| Colado2009 [26] | America | RT1 | 24Weeks | 2-3days | 35-60min | N/A | 15 | 54.70 ± 2.00 | BMI, WC, TC, HDL-C, LDL-C, TG, Glu, SBP, DBP |
|  |  | RT2 |  |  | 35-60min | N/A | 21 | 54.00 ± 2.80 |  |
|  |  | CON |  |  | N/A | N/A | 10 | 52.90 ± 1.90 |  |
| Conceicao2013 [27] | Brazil | RT | 16Weeks | 3days | N/A | N/A | 10 | 53.40 ± 3.95 | BW, HDL-C, TG, WC, DBP, SBP, BMI, BF% |
|  |  | CON |  |  | N/A | N/A | 10 | 53.00 ± 5.70 |  |
| Correa2014 [28] | Brazil | RT1 | 12Weeks | 5days | 20min | N/A | 12 | 59.50 ± 6.30 | BW, BMI, WC, Glu, TC, BF% |
|  |  | RT2 |  |  | 40min | N/A | 11 |  |  |
|  |  | CON |  |  | N/A | N/A | 12 |  |  |
| Correa2015 [29] | Brazil | RT1 | 11Weeks | 3days | 15min | N/A | 13 | 58.90 ± 5.80 | BW, BF%, BMI, WC |
|  |  | RT2 |  |  | 45min | N/A | 12 |  |  |
|  |  | CON |  |  | N/A | N/A | 11 |  |  |
| Cuff2003 [30] | Canada | CT | 16Weeks | 3days | 75min | AE: 60-75% HRR  RT: N/A | 10 | 63.40 ± 2.20 | BW |
|  |  | AE |  |  | 75min | N/A | 9 | 59.40 ± 1.90 |  |
|  |  | CON |  |  | N/A | N/A | 9 | 60.00 ± 2.90 |  |
| Daneshyar2020 [31] | Iran | MBE | 8Weeks | 3days | 40-50min | RPE: 13 | 12 | 54.80 ± 4.20 | BW, BMI, BF% |
|  |  | CT |  |  | 60-70min | RPE: 13 | 12 | 55.30 ± 5.30 |  |
|  |  | CON |  |  | N/A | N/A | 9 | 55.70 ± 6.70 |  |
| Delshad2012 [32] | Iran | RT | 12Weeks | 3days | 60min | 80%-100% 1RM | 10 | 54.40 ± 4.70 | BW, BMI, BF% |
|  |  | CON |  |  | N/A | N/A | 10 | 56.70 ± 3.90 |  |
| Diniz2015 [33] | Brazil | AE | 8Weeks | N/A | 50min | 100% CV | 10 | 60.26 ± 8.51 | BW, Glu, TG, TC, HDL-C, |
|  |  | CON |  |  | N/A | N/A | 9 | 62.01 ± 7.83 | LDL-C, BF% |
| Duff2016 [34] | Canada | RT | 9Months | 3days | N/A | N/A | 22 | 65.30 ± 4.60 | BF% |
|  |  | CON |  |  | N/A | N/A | 22 | 65.00 ± 4.70 |  |
| Dupuit2022 [35] | France | CT | 12Weeks | 3days | N/A | AE: 85% HRmax  RT: N/A | 8 | 58.80 ± 5.30 | BMI, BW, WC, TC, HDL-C, LDL-C, TG |
|  |  | CON |  |  | N/A | N/A | 9 | 60.90 ± 4.80 |  |
| Egana2010 [36] | Ireland | RT | 12Weeks | 2days | 60min | 10RM | 8 | 69.00 ± 5.00 | BW, BMI, BF% |
|  |  | CON |  |  | N/A | N/A | 8 | 64.00 ± 4.00 |  |
| Elliott2002 [37] | United Kingdom | RT | 8Weeks | 3days | N/A | 80% 10RM | 8 | 58.00 ± 4.00 | TC, HDL-C, LDL-C, TG, BW, BMI, BF%, SBP, DBP |
|  |  | CON |  |  | N/A | N/A | 7 | 53.00 ± 3.00 |  |
| Englund2005 [38] | Sweden | RT | 12Months | 2days | 50min | N/A | 21 | 72.80 ± 3.60 | BW, BMI |
|  |  | CON |  |  | N/A | N/A | 19 | 73.20 ± 4.90 |  |
| Figueroa2011 [39] | America | CT | 6Months | 3days | 40min | AE, RT: 60% HRmax | 12 | 54.00 ± 2.00 | BW, BMI |
|  |  | CON |  |  | N/A | N/A | 12 | 54.00 ± 1.00 |  |
| Figueroa2014 [40] | America | MBE | 6Weeks | 3days | N/A | N/A | 15 | 56.00 ± 3.00 | BW, BMI, BF%, SBP, DBP |
|  |  | CON |  |  | N/A | N/A | 13 | 56.00 ± 3.00 |  |
| Figueroa2014 [41] | America | MBE | 12Weeks | 3days | N/A | N/A | 13 | 55.50 ± 0.70 | BW, BMI, BF%, SBP, DBP |
|  |  | CON |  |  | N/A | N/A | 12 | 56.40 ± 1.00 |  |
| Figueroa2015 [42] | America | MBE1 | 12Weeks | 3days | N/A | N/A | 12 | 56.00 ± 1.00 | BW, BMI, SDP |
|  |  | MBE2 |  |  | N/A | N/A | 12 | 58.00 ± 1.00 |  |
|  |  | CON |  |  | N/A | N/A | 12 | 58.00 ± 1.00 |  |
| Frank2005 [43] | America | AE | 12Months | 5days | 45min | 60%-75% HRmax | 87 | 60.70 ± 6.70 | Leptin, Glu, TG |
|  |  | CON |  | 1day | 45min | N/A | 86 | 60.60 ± 6.80 |  |
| Friedenreich2011 [44] | Canada | AE | 12Months | 5days | 45min | 70%-80% HRR | 160 | 61.20 ± 5.40 | Glu, Leptin, ADPN, BMI |
|  |  | CON |  |  | N/A | N/A | 160 | 60.60 ± 5.70 |  |
| Friedenreich2011 [45] | Canada | AE | 12Months | 5days | 45min | 70%-80% HRR | 160 | 61.20 ± 5.40 | BW, BMI, WC |
|  |  | CON |  |  | N/A | N/A | 160 | 60.60 ±5.70 |  |
| Gerage2013 [46] | Brazil | RT | 12Weeks | 3days | N/A | N/A | 15 | 65.50 ± 5.00 | BW, BMI, BF%, SBP, DBP |
|  |  | MBE |  | 2days | 25-30min | N/A | 14 | 66.20 ± 4.10 |  |
| Ghanbari_Niaki2018 [47] | Iran | RT | 8Weeks | 3days | N/A | 55% 1RM | 12 | 58.03 ± 4.70 | BW, BMI, BF% |
|  |  | CON |  |  | N/A | N/A | 12 | 56.50 ± 4.20 |  |
| Gomez_Tomas2018 [48] | Spain | RT | 12Weeks | 3days | 50min | N/A | 18 | 70.89 ± 4.42 | BW, WC, TC, HDL-C, LDL-C, TG |
|  |  | CON |  |  | N/A | N/A | 20 | 70.45 ± 5.44 |  |
| Guzel2022 [49] | Turkey | AE | 10Weeks | 5day | 40min | 50%-70% HRmax | 12 | 55.67 ± 3.44 | BW, BMI, BF%, WC, SBP, Glu, Leptin, ADPN |
|  |  | CON |  | 3day | N/A | N/A | 12 | 54.42 ± 4.01 |  |
| Ha2021 [50] | Japan | AE | 16Weeks | N/A | N/A | N/A | 6 | 75.83 ± 4.26 | TG, HDL-C, Glu, SBP, DBP, BMI, BF%, WC |
|  |  | CON |  | N/A | N/A | N/A | 7 | 76.71 ±5.91 |  |
| Habermann2015 [51] | Germany | AE | 12Months | 5days | 45min | 60%-85% HRmax | 117 | 59.10 ± 5.10 | BW |
|  |  | CON |  | N/A | N/A | N/A | 87 | 57.10 ± 4.40 |  |
| He2022 [52] | China | AE1 | 12Weeks | 5days | 50min | 40% HRmax | 15 | 57.60 ± 3.20 | BMI, SBP, DBP, TC, HDL-C, LDL-C, TG |
|  |  | AE2 | 8Weeks | 3days | 40min | 70%-80% HRmax | 8 | 54.30 ± 6.40 |  |
|  |  | AE3 | 8Weeks | 3days | 66min | 85%-95% HRmax | 10 | 55.80 ± 5.60 |  |
|  |  | CON | 12Weeks | N/A | N/A | N/A | 15 | 58.33 ± 3.06 |  |
| Heli2013 [53] | Finland | CT | 16Weeks | 2days | 65-70min | AE: 50%-80% HRmax  RT: 5%-10 % BW | 6 | 63.30 ± 9.60 | BW, BMI, WC, Glu, TC, HDL-C, LDL-C, TG |
|  |  | CON |  |  | N/A | N/A | 4 | 55.80 ± 6.10 |  |
| Henriquez2017 [54] | Chile | AE | 6Months | 3days | 40min | 60%-65% VO2max | 18 | 58 | BW, WC, SBP, DBP, TC, HDL-C, LDL-C, TG |
|  |  | RT |  |  | 40min | 20% 10RM | 16 | 55 |  |
| Hintze2018 [55] | Canada | RT | 12Months | 3days | N/A | 70%-80% 1RM | 25 | N/A | BW, BMI |
|  |  | CON |  |  | N/A | N/A | 29 | N/A |  |
| Irwin2003 [56] | America | AE | 12Months | 5days | 45min | 60%-75% HRmax | 87 | 61 | BW, BMI, WC |
|  |  | CON |  | 1day | 45min | N/A | 86 | 60.6 |  |
| Jahangard2009 [57] | Iran | AE | 3Weeks | 3days | 35min | 70% HRmax | 10 | 50.00 ± 2.00 | BW, BMI, BF%, SBP, DBP |
|  |  | CON |  |  | N/A | N/A | 10 | 50.00 ± 2.00 |  |
| Jaime2019 [58] | America | RT | 12Weeks | N/A | 20-35min | N/A | 12 | 64.00 ± 1.00 | SBP, DBP |
|  |  | MBE |  |  | 20-35min | N/A | 13 | 64.00 ± 1.00 |  |
|  |  | CON |  |  | N/A | N/A | 8 | 67.00 ± 1.00 |  |
| Jamka2021 [59] | Poland | AE | 12Weeks | 3days | 60min | 50%-75% HRmax | 52 | 55.00 ± 7.00 | SBP, DBP |
|  |  | CT |  |  | 60min | AE: 50%-75% HRmax  RT: 50%-60% HRmax | 49 | 55.00 ± 7.00 |  |
| Jeon2020 [60] | Korea | CT | 12Weeks | 3days | 50min | AE: RPE 11-14  RT: RPE 13-14 | 30 | 62.10 ± 7.30 | SBP, DBP, LDL-C, HDL-C, TC, TG, BW, WC, BMI, BF% |
|  |  | CON |  |  | N/A | N/A | 15 | 61.10 ± 7.00 |  |
| Jo2020 [61] | Korea | MBE | 12Weeks | 2days | 50min | 42%-82% HRR | 22 | 61.80 ± 10.10 | BW, BMI, WC, SBP, DBP |
|  |  | AE |  |  | 50min | 60%-80% HRR | 22 | 57.30 ± 8.40 |  |
|  |  | CON |  |  | N/A | N/A | 21 | 28.60 ± 12.70 |  |
| Johannsen2012 [62] | America | AE1 | 21Weeks | N/A | N/A | N/A | 134 | 58.10 ± 6.60 | BW, BMI, WC, Glu, ADPN |
|  |  | AE2 |  |  | N/A | N/A | 78 | 57.20 ± 6.80 |  |
|  |  | AE3 |  |  | N/A | N/A | 92 | 56.60 ± 6.50 |  |
|  |  | CON |  |  | N/A | N/A | 86 | 57.10 ± 5.70 |  |
| Kang2009 [63] | Korea | RT | 12Weeks | 3days | 60min | 60% HRR | 8 | 50.40 ± 2.14 | BW, BMI, BF%, Glu |
|  |  | AE |  | 3days | 60min | 60% HRR | 7 | 52.50 ± 2.15 |  |
| Kazemi2023 [64] | Iran | RT | 8Weeks | 3day | N/A | 75% 1RM | 15 | 45.00-65.00 | BW, BMI, WC, HDL-C, LDL-C, TG, SBP, DBP |
|  |  | AE |  |  | N/A | 80%-90% HRmax | 15 |  |  |
|  |  | CON |  |  | N/A | N/A | 15 |  |  |
| Keawtep2024 [65] | Thailand | AE | 3Months | 3day | 60min | N/A | 23 | 52.70 ± 3.60 | TC, TG, Glu |
|  |  | CON |  | 3day | N/A | N/A | 23 | 53.61 ± 2.81 |  |
| Kemmler2009 [66] | Germany | CT | 12Months | 4days | 60min | N/A | 33 | 68.70 ±3.40 | WC, TG, HDL-C, SBP, DBP, Glu |
|  |  | CON |  | 1day | 60min | N/A | 32 | 69.50 ± 4.30 |  |
| Khalid2013 [67] | Libya | AE | 8Weeks | 3days | 20min | 60%-70% HRmax | 15 | 52.90 ± 2.60 | BMI, SBP, DBP |
|  |  | CON |  |  | N/A | N/A | 15 | 52.70 ± 2.20 |  |
| Kheirat2018 [68] | Algeria | AE | 12Months | 3days | 60min | 60% HRmax | 30 | 55.00 ± 4.00 | Glu, TC, TG, LDL-C, HDL-C |
|  |  | CON |  |  | N/A | N/A | 35 | 56.00 ± 2.00 |  |
| Kim2012 [69] | Korea | AE | 16Weeks | 3days | 60min | 55%-80% HRmax | 15 | 54.53 ± 2.82 | BF%, BW, BMI, WC, TG, HDL-C, LDL-C, SBP, DBP, TC, Glu |
|  |  | CON |  |  | N/A | N/A | 15 | 54.53 ± 2.82 |  |
| Kim2021 [70] | Korea | AE | 12Weeks | 3days | 60min | RPE: 12-15 | 12 | 74.36 ± 3.78 | BF%, WC, HDL-C, LDL-C |
|  |  | CON |  |  | N/A | N/A | 10 | 75.90 ± 4.23 |  |
| Klentro2007 [71] | Canada | RT | 12Weeks | 3days | 65min | 75% HRmax | 9 | 52.70 ± 4.10 | BW, BF% |
|  |  | CON |  |  | N/A | N/A | 7 | 53.40 ± 5.60 |  |
| Kobayashi2022 [72] | Japan | AE1 | 8Weeks | 2days | 35min | 65% HRmax | 15 | 62.50 ± 2.80 | BW, BMI, SBP, DBP |
|  |  | AE2 |  | 2days | 35min | 65% HRmax | 15 | 62.90 ± 1.90 |  |
|  |  | CON |  | N/A | N/A | N/A | 15 | 60.90 ± 3.90 |  |
| Koch2021 [73] | Brazil | MBE | 10Weeks | 2-4days | 45-55min | N/A | 20 | 64.20 | TC, TG, Glu |
|  |  | CON |  |  | N/A | N/A | 6 | 64.16 |  |
| Latosik2014 [74] | Poland | AE | 8Weeks | N/A | 65-67min | 40%-69% HRmax | 15 | N/A | BW, BMI, WC, SBP, DBP, TC, TG |
|  |  | CON |  |  | N/A | N/A | 10 |  |  |
| Lee2012 [75] | Korea | MBE | 16Weeks | 3days | 60min | N/A | 8 | 54.75 ± 2.76 | BW, BMI, WC, ADPN, TC, TG, HDL-C, LDL-C, SBP, DBP, Glu |
|  |  | CON |  |  | N/A | N/A | 8 | 54.25 ± 2.91 |  |
| Lee2017 [76] | Korea | AE | 16Weeks | 3days | 60min | 50%-80% HRmax | 18 | 55.00 ± 1.86 | BW, BF%, BMI, WC, Leptin, Glu, TC, TG, HDL-C, LDL-C, SBP, DBP |
|  |  | CON |  |  | N/A | N/A | 18 | 54.10 ± 1.95 |  |
| LeeYong2021 [77] | Korea | MBE | 16Weeks | 5days | 60min | 50%-80% HRmax | 12 | 56.00 ± 2.90 | BW, BMI, TC, TG, LDL-C, HDL-C |
|  |  | CON |  |  | N/A | N/A | 12 | 57.50 ± 2.90 |  |
| Leon2018 [78] | Korea | CT | 12Weeks | 3days | 60min | AE: 65%-80% HRmax  RT: N/A | 8 | 59.00 ± 1.00 | BW, BMI, SBP, DBP, Glu |
|  |  | CON |  |  | N/A | N/A | 8 | 58.00 ± 2.00 |  |
| Maesta2007 [79] | Brazil | RT | 16Weeks | 3days | 40-50min | 60-80% 1RM | 11 | 60.70 ± 7.10 | BMI, WC, BF%, TC, TG |
|  |  | CON |  |  | N/A | N/A | 11 | 57.90 ± 6.90 |  |
| Martins2018 [80] | Brazil | AE | 12Weeks | 3days | 60min | >85% HRmax Borg 7-8 | 8 | 64.30 ± 6.70 | BF%, Glu |
|  |  | CT |  |  | 60min | AE: >70 HRmax  RT: 70% 1RM | 8 | 65.00 ± 6.30 |  |
| McGavock2004 [81] | Canada | CT | 10Weeks | 3days | N/A | AE: 65%-75% HRR  RT: 65%-70% 1RM | 11 | 58.00 ± 7.00 | BW, BMI, TC, SBP, DBP |
|  |  | CON |  |  | N/A | N/A | 7 | 59.00 ± 5.00 |  |
| Miura2015 [82] | Japan | CT1 | 12Weeks | 2days | 90min | N/A | 58 | 72.00 ± 7.10 | BW, BF%, SBP, DBP |
|  |  | CON1 |  |  | N/A | N/A | 57 | 71.80 ± 5.60 |  |
|  |  | CT2 |  |  | 90min | N/A | 53 | 72.90 ± 5.70 |  |
|  |  | CON2 |  |  | N/A | N/A | 53 | 69.70 ± 6.70 |  |
| Miyaki2012 [83] | Japan | AE | 8Weeks | 3-5days | 30-45min | 70%-80% HRmax | 10 | 60.00 ± 6.00 | BW, BMI, TC, TG, HDL-C, LDL-C, SBP, DBP |
|  |  | CON |  |  | N/A | N/A | 10 | 60.00 ± 7.00 |  |
| Mohanka2006 [84] | America | CT | 12Months | 5days | 45min | AE, RT: 60%-75% HRmax | 87 | 60.60 ± 6.60 | TC, TG, LDL-C, HDL-C |
|  |  | CON |  | 1day | 60min | N/A | 86 | 60.50 ± 6.70 |  |
| Nazarabadi2022 [85] | Iran | AE | 8Weeks | 3days | 45-6min | 60-%75% HRmax | 12 | 45.25 ± 2.22 | BW, BMI, WC, TG, Glu |
|  |  | CON |  |  | N/A | N/A | 11 | 56.36 ± 1.91 |  |
| Neves2017 [86] | Brazil | CT | 16Weeks | 3days | 55-56.5min | N/A | 28 | 58.60 ± 3.90 | BW, BMI, Glu, TC, HDL-C, LDL-C, BF% |
|  |  | CON |  |  | N/A | N/A | 22 | 57.70 ± 4.80 |  |
| Nishida2015 [87] | Japan | AE | 12Weeks | 3days | 46min | N/A | 31 | 70.40 ± 5.80 | BW, BMI, SBP, DBP, TC, HDL-C, TG, LDL-C |
|  |  | CON |  |  | N/A | N/A | 31 | 69.70 ± 6.60 |  |
| Nunes2016 [88] | Brazil | RT1 | 16Weeks | 3days | N/A | 70% 1RM | 10 | 62.00 | TC, HDL-C, LDL-C, TG, WC |
|  |  | RT2 |  |  | N/A | 70% 1RM | 11 | 62.00 |  |
|  |  | CON |  |  | N/A | N/A | 11 | 60.00 |  |
| Nunes2017 [89] | Brazil | RT1 | 16Weeks | 3days | N/A | 70% 1RM | 10 | 64.20 | BW, WC |
|  |  | RT2 |  |  | N/A | 70% 1RM | 12 | 59.70 |  |
|  |  | CON |  |  | N/A | N/A | 11 | 59.40 |  |
| Nunes2019 [90] | Brazil | AE | 12Weeks | 3days | 28min | >80% HRmax | 13 | 62.90 | BW, BMI, ADPN, Leptin |
|  |  | CT |  |  | 60min | AE: 70% HRmax  RT: 70% 1RM | 13 | 62.30 |  |
| Nunes2022 [91] | Brazil | AE | 12Weeks | 3days | 30min | >80% HRmax | 24 | 61.50 ± 7.00 | SBP, DBP |
|  |  | CT |  |  | 30min | AE:70% HRmax  RT:70% 1RM | 25 | 62.60 ± 8.50 |  |
| Orsatti2008 [92] | Brazil | RT | 16Weeks | 3days | 50-60min | 60-80% 1RM | 22 | 57.80 ± 8.00 | BMI, WC, BF% |
|  |  | CON |  |  | N/A | N/A | 21 | 59.30 ± 6.20 |  |
| Paolillo2013 [93] | Brazil | MBE | 6Months | 2days | 45min | 85%-90% HRmax | 10 | 55.00 ± 2.00 | SBP, DBP |
|  |  | CON |  |  | N/A | N/A | 10 | 55.00 ± 2.00 |  |
| Paolillo2017 [94] | Brazil | AE | 6Months | 2days | 45min | 85%-90% HRmax | 10 | 50.00-60.00 | BW, WC, TG, TC, BF% |
|  |  | CON |  |  | N/A | N/A | 10 |  |  |
| Park2015 [95] | Korea | CT | 12Weeks | 3days | 70min | RT: 60%-70 1RM  AE: 40%-75% HRmax | 10 | 57.20 ± 2.57 | BW, BF%, HDL-C, LDL-C, TC, TG |
|  |  | CON |  |  | N/A | N/A | 10 | 57.20 ± 1.69 |  |
| Pereira2021 [96] | Portugal | MBE | 16Weeks | 2-3days | 60min | N/A | 41 | 67.30 ± 6.50 | BW, BF% |
|  |  | CON |  |  | N/A | N/A | 26 | 69.90 ± 5.40 |  |
| Pereira2023 [97] | Portugal | MBE | 36Weeks | 2-3days | 60min | 77%-79% HRmax | 31 | 64.00 ± 7.00 | Glu, TC, HDL-C, LDL-C, TG, BW, BF% |
|  |  | CON |  |  | N/A | N/A | 14 | 67.00 ± 5.00 |  |
| Perez2022 [98] | Spain | AE | 12Weeks | 3days | 60min | 55-75% HRR | 10 | 56.70 ± 3.70 | BW, BMI, Glu, TG |
|  |  | CT |  |  | 60min | AE: 55%-75% HRR  RT: 65% 1RM | 13 | 58.70 ± 2.90 |  |
|  |  | CON |  |  | N/A | N/A | 12 | 56.90 ± 5.80 |  |
| Phillips2012 [99] | America | RT | 12Weeks | 3days | 75min | N/A | 11 | 64.80 ± 2.40 | BW, BF%, BMI |
|  |  | CON |  |  | N/A | N/A | 12 | 66.40 ± 2.80 |  |
| pospieszna2017 [100] | Poland | AE | 12Weeks | 3days | 60min | N/A | 20 | 62.00 ± 3.79 | BW, BMI, SBP, DBP, Glu, TG, HDL-C, LDL-C, TC |
|  |  | CON |  |  | N/A | N/A | 19 | 62.00 ± 1.10 |  |
| Quintao2018 [101] | Brazil | CT | 20Weeks | 3days | 30min | RT: 70%-85% 1RM | 25 | 69.44 ± 6.82 | BW, BMI |
|  |  | CON |  |  | N/A | N/A | 26 | 68.30 ± 6.34 |  |
| Rashti2019 [102] | Iran | CT1 | 10Weeks | 3days | 50-65min | AE: 85%-95% HRmax  RT: 60%-85% 1RM | 15 | 57.11 ± 4.13 | BW, BMI, Glu |
|  |  | CT2 |  |  | 50-65min | AE: 60%-75% HRmax  RT: 40%-70% 1RM | 14 | 54.10 ± 5.08 |  |
|  |  | CON |  |  | N/A | N/A | 10 | 54.10 ± 5.08 |  |
| Ready1996 [103] | Canada | AE1 | 24Weeks | 3days | 60min | 60% VO2 | 19 | >50.00 | BW, BMI, LDL-C, HDL-C, TG, |
|  |  | AE2 |  | 5days | 60min | 60% VO2 | 17 |  |  |
|  |  | CON |  | N/A | N/A | N/A | 20 |  |  |
| Reis2012 [104] | Brazil | RT | 3Months | 2days | N/A | 60%-85% 1RM | 27 | 52.91 ± 4.02 | SBP, DBP |
|  |  | CON |  |  | N/A | N/A | 31 | 53.86 ± 5.11 |  |
| Rossi2016 [105] | Brazil | AE | 16Weeks | 3days | 52min | 100% LAN | 15 | 61.00 ± 6.30 | BW, BMI, TC, LDL-C, HDL-C, BF% |
|  |  | CT |  |  | 57min | RT:65% 1RM  AE:100% LAN | 32 |  |  |
|  |  | CON |  |  | N/A | N/A | 18 |  |  |
| Rossi2017 [106] | Brazil | AE | 16Weeks | 3days | 50min | 100% LAN | 20 | 60.60 ± 7.90 | BW, BF%, Glu, TG, TC, HDL-C, LDL-C, Leptin, ADPN |
|  |  | CT |  |  | 60min | AE: 100% LAN  RT: N/A | 20 | 62.40 ± 5.10 |  |
|  |  | CON |  |  | N/A | N/A | 20 | 62.80 ± 5.90 |  |
| Rossi2017 [107] | Brazil | CT1 | 8Weeks | 3days | 70min | RT: 65%-75% 1RM  AE: 100% CV | 20 | 62.20 ± 6.30 | BW, Glu, TG, TC, HDL-C, LDL-C, BF% |
|  |  | CT2 |  |  | 67-70min | FT: RPE: 12-13 AE: 100% CV | 17 | 60.10 ± 5.40 |  |
|  |  | CON |  |  | N/A | N/A | 15 | 61.90 ± 7.20 |  |
| Rossi2018 [108] | Brazil | AE | 16Weeks | 3days | 50min | N/A | 16 | 61.40 ± 5.00 | LDL-C, HDL-C, TC, Glu, TG |
|  |  | CT |  |  | 60min | N/A | 13 | 61.40 ± 5.00 |  |
|  |  | CON |  |  | N/A | N/A | 10 | 63.40 ± 7.50 |  |
| Saeidi2019 [109] | Iran | RT | 8Weeks | 3days | N/A | 55% 1RM | 12 | 58.00 ± 5.00 | Leptin |
|  |  | CON |  |  | N/A | N/A | 12 | 56.00 ± 5.00 |  |
| Seo2012 [110] | Korea | CT | 12Weeks | 3days | N/A | AE, RT: 60% HRmax | 8 | 54.80 ± 7.70 | BW, BF%, BMI, HDL-C, TG, SBP, DBP |
|  |  | CON |  |  | N/A | N/A | 6 | 54.40 ± 5.40 |  |
| Serrano2016 [111] | Spain | MBE | 8Weeks | 3days | 50min | N/A | 27 | 69.07 ± 4.41 | SBP, DBP, BMI |
|  |  | CON |  |  | N/A | N/A | 25 | 69.48 ± 3.22 |  |
| Shabani2018 [112] | Iran | CT | 8Weeks | 3days | 90min | AE: 50%-80% HRmax  RT: 50%-75% 1RM | 12 | 54.83 ± 4.72 | BW, BMI, SBP, DBP, TG |
|  |  | CON |  |  | N/A | N/A | 12 | 56.90 ± 4.93 |  |
| Shaw2016 [113] | Republic of South Africa | RT | 6Weeks | 2days | 40min | 67%-85% 1RM | 19 | 60.44 ± 5.34 | SBP, DBP, Glu, TC, BW, BMI, WC |
|  |  | CON |  |  | N/A | N/A | 18 | 57.74 ± 2.83 |  |
| Shen2013 [114] | China | AE | 10Weeks | 3days | 90min | 75%-85% HRR | 32 | 57.86 ± 0.64 | BW, BMI, TC, LDL-C, TG, HDL-C, Glu, BF% |
|  |  | CON |  |  | N/A | N/A | 30 | 59.10 ± 0.83 |  |
| Shiotsu2018 [115] | Japan | CT1 | 10Weeks | 2days | 40min | AE: 60% HRR RT: 60%-70% 1RM | 12 | 68.30 ± 4.20 | BW, BMI, BF%, WC |
|  |  | CT2 |  |  | 40min | AE: 60% HRR RT: 60%-70% 1RM | 12 | 69.00 ± 4.10 |  |
|  |  | CT3 |  |  | 40min | AE:60% HRR RT: 60%-70% 1RM | 12 | 70.40 ± 4.10 |  |
|  |  | CT4 |  |  | 40min | AE: 60% HRR RT: 60%-70% 1RM | 12 | 69.60 ± 4.60 |  |
|  |  | CON |  |  | N/A | N/A | 12 | 71.00 ± 4.40 |  |
| Sobczak2023 [116] | Poland | AE | 8Weeks | 2days | 65-70min | 50%-70% HRR | 16 | 65.04 ± 4.01 | BW, BMI, WC, SBP, DBP |
|  |  | CT |  |  | 65-70min | 50%-70% HRR | 16 | 67.62 ± 4.29 |  |
| Sobrinho2023 [117] | Brazil | MBE1 | 14Weeks | 2days | N/A | N/A | 23 | 64.40 ± 2.30 | BW, SBP, DBP |
|  |  | MBE2 |  |  | N/A | N/A | 28 | 65.10 ± 4.00 |  |
|  |  | CT1 |  |  | 90min | N/A | 20 | 65.30 ± 5.00 |  |
|  |  | CT2 |  |  | 90min | N/A | 23 | 65.30 ± 3.90 |  |
|  |  | CON1 |  |  | N/A | N/A | 21 | 66.10 ± 4.90 |  |
|  |  | CON2 |  |  | N/A | N/A | 26 | 66.80 ± 5.40 |  |
| Son2020 [118] | Korea | RT | 12Weeks | 3days | 60min | 40%-70% 1RM | 20 | 67.70 ± 1.00 | BW, BMI, BF%, SBP, DBP |
|  |  | CON |  |  | N/A | N/A | 10 | 67.40 ± 1.10 |  |
| Son2023 [119] | Korea. | AE | 12Weeks | N/A | N/A | 64%-76% HRmax | 14 | 70.20 ± 1.21 | BW, BMI, BF% |
|  |  | CON |  | N/A | N/A | N/A | 12 | 69.90 ± 1.14 |  |
| SonWon2017 [120] | Korea | CT | 12Weeks | 3days | 60min | AE-RT: 40%-70% HRR | 10 | 76.00 ± 5.00 | BW, BMI, BF% |
|  |  | CON |  |  | N/A | N/A | 10 | 74.70 ± 2.00 |  |
| Soori2017 [121] | Iran | AE | 10Weeks | 3days | 45min | 40-60 HRmax | 8 | 45.00-60.00 | BW, BMI, TC, TG, HDL-C, LDL-C |
|  |  | RT |  |  | 45min | 40-60 1RM | 8 |  |  |
|  |  | CT |  |  | 44min | AE: 40-60 HRmax  RT: 40-60 1RM | 8 |  |  |
|  |  | CON |  |  | N/A | N/A | 8 |  |  |
| Sugawara2012 [122] | Japan | AE | 8Weeks | 3-6days | 25-45min | 60%-75% HRmax | 11 | 59.00 ± 2.00 | BW, BMI, SBP, DBP, LDL-C, HDL-C |
|  |  | CON |  |  | N/A | N/A | 11 | 59.00 ± 2.00 |  |
| Taha2016 [123] | Egypt | AE | 10Weeks | 3days | 40min | 70%-85% HRmax | 23 | 48.17 ± 2.20 | BMI, SBP, DBP |
|  |  | CON |  |  | N/A | N/A | 23 | 47.78 ± 2.59 |  |
| Takahashi2013 [124] | Japan | AE | 12Weeks | 2days | 30-60min | N/A | 6 | 72.00 ± 2.10 | BW, BMI, WC, SBP, DBP |
|  |  | CON |  |  | N/A | N/A | 8 | 68.40 ± 1.40 |  |
| Tan2018 [125] | China | AE | 12Weeks | 3days | 60min | N/A | 16 | 63.00 ± 2.30 | BW, BMI, BF%, WC, ADPN, Leptin, TC, TG, HDL-C, LDL-C |
|  |  | CON |  |  | N/A | N/A | 15 | 62.90 ± 2.60 |  |
| Tanahashi2014 [126] | Japan | AE | 12Weeks | 3-6days | 40-60min | 65-80% HRmax | 20 | 62.00 ± 6.00 | BW, BMI, SBP, DBP |
|  |  | CON |  |  | N/A | N/A | 10 | 61.00 ± 7.00 |  |
| Tapp2014 [127] | America | MBE | 8Weeks | 3days | 17-20min | N/A | 6 | 53.20 ± 2.10 | BW, BMI, BF% |
|  |  | AE |  |  | 45min | 40% HRmax | 6 | 55.20 ± 6.40 |  |
|  |  | RT |  |  | 45min | 55%-60% 1RM | 7 | 54.10 ± 5.30 |  |
| Tayebi2019 [128] | Iran | RT1 | 8Weeks | 3days | 20min | 35% 1RM | 12 | 57.70 ± 3.64 | BW, BMI, BF% |
|  |  | RT2 |  |  | 20min | 55% 1RM | 12 | 58.00 ± 4.70 |  |
|  |  | CON |  |  | N/A | N/A | 12 | 56.50 ± 4.20 |  |
| Trabka2014 [129] | Poland. | CT | 10Weeks | 2days | 80min | 50%-80% HRmax | 23 | N/A | BW, BMI, WC, TC, TG, HDL-C, LDL-C |
|  |  | CON |  |  | N/A | N/A | 21 | N/A |  |
| Ward2020 [130] | Sweden | RT | 15Weeks | 3days | N/A | N/A | 26 | 55.70 ± 5.10 | BW, BMI, ADPN, Leptin |
|  |  | CON |  |  | N/A | N/A | 29 | 55.40 ± 5.00 |  |
| Wen2017 [131] | China | AE | 10Weeks | 3days | 90min | 75%-85% HRR | 24 | 57.50 ± 3.50 | BW, BMI, BF%, TC, TG |
|  |  | CON |  |  | N/A | N/A | 22 | 58.80 ± 3.20 |  |
| Wieczorek2012 [132] | Poland | AE | 8Weeks | 3days | 40min | 70% ~ 80% CV | 27 | 64.00 ± 6.13 | BW, BMI, Glu |
|  |  | CON |  |  | N/A | N/A | 17 | 65.00 ± 7.43 |  |
| Wong2014 [133] | America | RT | 8Weeks | 3days | 50min | 70% 1RM | 14 | 57.00 ± 1.00 | BW, BMI, SBP, DBP |
|  |  | CON |  |  | N/A | N/A | 14 | 56.00 ± 1.00 |  |
| Wong2016 [134] | America | MBE | 8Weeks | 3days | 11-60min | N/A | 13 | 58.00 ± 1.00 | BW, BMI, SBP, DBP |
|  |  | CON |  |  | N/A | N/A | 12 | 59.00 ± 1.00 |  |
| Wong2018 [135] | America | AE | 12Weeks | 4days | N/A | RPE 11-13 | 20 | 59.00 ± 1.00 | BW, BMI, SBP, DBP |
|  |  | CON |  |  | N/A | N/A | 21 |  |  |
| WongAlexei2018 [136] | America | AE | 20Weeks | 3-4days | 50-45min | 60%-75% HRmax | 52 | 74.00 ± 4.00 | BW, BMI, BF%, SBP, DBP |
|  |  | CON |  |  | N/A | N/A | 48 | 73.00 ± 4.00 |  |
| Wooten2011 [137] | America | RT | 12Weeks | 3days | N/A | 50% 1RM | 9 | 65.90 ± 0.50 | TG, TC, LDL-C, HDL-C |
|  |  | CON |  |  | N/A | N/A | 12 | 67.00 ± 0.60 |  |
| Wu2006 [138] | Japan | AE | 24Weeks | 3days | 60min | N/A | 31 | 55.20 ± 2.80 | BW, BMI, TC, TG, HDL-C, LDL-C |
|  |  | CON |  |  | N/A | N/A | 33 | 54.90 ± 2.90 |  |
| Yoon2018 [139] | Korea | AE | 12Weeks | 3days | 60min | 60%-80% HRR | 10 | 53.70 ± 3.37 | BW, BMI, BF%, WC, SBP, DBP, TG, HDL-C, LDL-C |
|  |  | RT |  |  | 60min | 60% 1RM | 10 | 52.20 ± 2.15 |  |
|  |  | CON |  |  | N/A | N/A | 10 | 52.50 ± 2.68 |  |
| Yoshizawa2009 [140] | Japan | AE | 8Weeks | 3-5days | 25-45min | 60%-75% HRmax | 12 | 57.00 ± 1.00 | BMI, BW, TC, HDL-C, LDL-C, TG, SBP, DBP |
|  |  | CON |  |  | N/A | N/A | 13 | 59.00 ± 1.00 |  |
| Yoshizawa2010 [141] | Japan | AE | 8Weeks | 3-5days | 25-45min | 60%-75% HRmax | 10 | 57.00 ± 1.00 | BMI, BW, TC, HDL-C, LDL-C, TG, SBP, DBP |
|  |  | CON |  |  | N/A | N/A | 10 | 58.00 ± 1.00 |  |
| Zhang2019 [142] | Australia | AE | 8Weeks | 3days | 30min | 80%-85% HRmax | 15 | 53.20 ± 3.50 | BW, BMI, SBP, DBP |
|  |  | CON |  |  | N/A | N/A | 15 | 53.00 ± 3.10 |  |
| ADPN: adiponectin; AE: aerobic exercise; BF: body fat; BMI: body mass index; BW: body weight; CON: control; CT: combined training; CV: critical velocity; DBP: diastolic blood pressure; Glu: glucose; HDL-C: high-density lipoprotein cholesterol; HRmax: Maximal Heart Rate; HRR: heart rate reserve; LDL-C: low-density lipoprotein cholesterol; MBE: mind-body exercise; RPE: rating of perceived exertion; RT: resistance training; SBP: systolic blood pressure; TC: total cholesterol; TG: triglyceride; WC: waist circumference; 1RM: one-repetition maximum. | | | | | | | | | |

# Table S3 CINeMA Results

| **Comparison** | **Number of studies** | **Within-study bias** | **Reporting bias** | **Indirectness** | **Imprecision** | **Heterogeneity** | **Incoherence** | **Confidence rating** |
| --- | --- | --- | --- | --- | --- | --- | --- | --- |
| **BW** | | | | | | | | |
| AE:CON | 50 | Some concerns | Low risk | No concerns | No concerns | Major concerns | No concerns | Low |
| AE:CT | 9 | Some concerns | Low risk | No concerns | Some concerns | Some concerns | No concerns | Low |
| AE:MBE | 2 | Some concerns | Low risk | No concerns | Major concerns | No concerns | No concerns | Low |
| AE:RT | 6 | Some concerns | Low risk | No concerns | Some concerns | Some concerns | No concerns | Low |
| CON:CT | 25 | Some concerns | Low risk | No concerns | No concerns | Some concerns | No concerns | Moderate |
| CON:MBE | 9 | Some concerns | Low risk | No concerns | Major concerns | No concerns | No concerns | Low |
| CON:RT | 24 | Some concerns | Low risk | No concerns | Some concerns | Some concerns | No concerns | Low |
| CT:MBE | 1 | Some concerns | Low risk | No concerns | Some concerns | Some concerns | No concerns | Low |
| CT:RT | 1 | Some concerns | Low risk | No concerns | Some concerns | Some concerns | No concerns | Low |
| MBE:RT | 2 | Some concerns | Low risk | No concerns | Major concerns | No concerns | No concerns | Low |
| **BF%** | | | | | | | | |
| AE:CON | 24 | Some concerns | Low risk | No concerns | No concerns | Some concerns | No concerns | Moderate |
| AE:CT | 5 | Some concerns | Low risk | No concerns | No concerns | Some concerns | No concerns | Moderate |
| AE:MBE | 1 | Some concerns | Low risk | No concerns | Some concerns | No concerns | No concerns | Moderate |
| AE:RT | 4 | Some concerns | Low risk | No concerns | No concerns | Some concerns | No concerns | Moderate |
| CON:CT | 14 | Some concerns | Low risk | No concerns | No concerns | Some concerns | No concerns | Moderate |
| CON:MBE | 3 | Some concerns | Low risk | No concerns | No concerns | Major concerns | No concerns | Low |
| CON:RT | 17 | Some concerns | Low risk | No concerns | No concerns | Some concerns | No concerns | Moderate |
| CT:RT | 1 | Some concerns | Low risk | No concerns | No concerns | Major concerns | No concerns | Low |
| MBE:RT | 2 | Some concerns | Low risk | No concerns | No concerns | Some concerns | No concerns | High |
| CT:MBE | 0 | Some concerns | Low risk | No concerns | No concerns | Some concerns | No concerns | Moderate |
| **BMI** | | | | | | | | |
| AE:CON | 49 | Some concerns | Low risk | No concerns | No concerns | Major concerns | No concerns | Low |
| AE:CT | 8 | Some concerns | Low risk | No concerns | Some concerns | Some concerns | No concerns | Moderate |
| AE:MBE | 2 | Some concerns | Low risk | No concerns | Major concerns | No concerns | No concerns | Low |
| AE:RT | 5 | Some concerns | Low risk | No concerns | Major concerns | No concerns | No concerns | Low |
| CON:CT | 21 | Some concerns | Low risk | No concerns | No concerns | Major concerns | No concerns | Low |
| CON:MBE | 9 | Some concerns | Low risk | No concerns | Major concerns | No concerns | No concerns | Low |
| CON:RT | 23 | Some concerns | Low risk | No concerns | No concerns | Major concerns | No concerns | Low |
| CT:RT | 1 | Some concerns | Low risk | No concerns | Major concerns | No concerns | No concerns | Low |
| MBE:RT | 2 | Some concerns | Low risk | No concerns | Major concerns | No concerns | No concerns | Low |
| CT:MBE | 0 | Some concerns | Low risk | No concerns | Some concerns | Some concerns | No concerns | Low |
| **WC** | | | | | | | | |
| AE:CON | 21 | Some concerns | Low risk | No concerns | No concerns | Major concerns | No concerns | Low |
| AE:CT | 1 | Some concerns | Low risk | No concerns | Major concerns | No concerns | No concerns | Low |
| AE:MBE | 1 | Major concerns | Low risk | No concerns | Major concerns | No concerns | No concerns | Low |
| AE:RT | 3 | Some concerns | Low risk | No concerns | Some concerns | Some concerns | No concerns | Low |
| CON:CT | 8 | Some concerns | Low risk | No concerns | Some concerns | Some concerns | No concerns | Low |
| CON:MBE | 2 | Major concerns | Low risk | No concerns | Major concerns | No concerns | No concerns | Low |
| CON:RT | 12 | Some concerns | Low risk | No concerns | No concerns | Major concerns | No concerns | Low |
| CT:MBE | 0 | Some concerns | Low risk | No concerns | Major concerns | No concerns | No concerns | Low |
| CT:RT | 0 | Some concerns | Low risk | No concerns | Some concerns | Some concerns | No concerns | Low |
| MBE:RT | 0 | Some concerns | Low risk | No concerns | Major concerns | No concerns | No concerns | Low |
| **DBP** | | | | | | | | |
| AE:CON | 31 | Some concerns | Low risk | No concerns | No concerns | Major concerns | No concerns | Low |
| AE:CT | 3 | Some concerns | Low risk | No concerns | Major concerns | No concerns | No concerns | Low |
| AE:MBE | 1 | Some concerns | Low risk | No concerns | No concerns | Major concerns | No concerns | Low |
| AE:RT | 4 | Some concerns | Low risk | No concerns | Major concerns | No concerns | No concerns | Low |
| CON:CT | 11 | Some concerns | Low risk | No concerns | No concerns | Major concerns | No concerns | Low |
| CON:MBE | 8 | Some concerns | Low risk | No concerns | No concerns | Some concerns | No concerns | Low |
| CON:RT | 11 | Some concerns | Low risk | No concerns | No concerns | Major concerns | No concerns | Low |
| CT:MBE | 1 | Some concerns | Low risk | No concerns | No concerns | Major concerns | Some concerns | Low |
| MBE:RT | 2 | Some concerns | Low risk | No concerns | Some concerns | Some concerns | Some concerns | Very low |
| CT:RT | 0 | Some concerns | Low risk | No concerns | Major concerns | No concerns | Major concerns | Very low |
| **SBP** | | | | | | | | |
| AE:CON | 32 | Some concerns | Low risk | No concerns | No concerns | Major concerns | No concerns | Low |
| AE:CT | 2 | Some concerns | Low risk | No concerns | Major concerns | No concerns | No concerns | Low |
| AE:MBE | 1 | Some concerns | Low risk | No concerns | Some concerns | Some concerns | No concerns | Low |
| AE:RT | 5 | Some concerns | Low risk | No concerns | Major concerns | No concerns | No concerns | Low |
| CON:CT | 11 | Some concerns | Low risk | No concerns | Some concerns | Some concerns | No concerns | Low |
| CON:MBE | 9 | Some concerns | Low risk | No concerns | No concerns | Major concerns | No concerns | Low |
| CON:RT | 11 | Some concerns | Low risk | No concerns | No concerns | Major concerns | No concerns | Low |
| CT:MBE | 1 | Some concerns | Low risk | No concerns | Some concerns | Some concerns | No concerns | Low |
| MBE:RT | 2 | Some concerns | Low risk | No concerns | Major concerns | No concerns | No concerns | Low |
| CT:RT | 0 | Some concerns | Low risk | No concerns | Major concerns | No concerns | No concerns | Low |
| **Glu** | | | | | | | | |
| AE:CON | 22 | Some concerns | Low risk | No concerns | No concerns | Major concerns | No concerns | Low |
| AE:CT | 5 | Some concerns | Low risk | No concerns | Major concerns | No concerns | No concerns | Low |
| AE:RT | 1 | Some concerns | Low risk | No concerns | Major concerns | No concerns | No concerns | Low |
| CON:CT | 10 | Some concerns | Low risk | No concerns | Major concerns | No concerns | No concerns | Low |
| CON:MBE | 3 | Major concerns | Low risk | No concerns | Major concerns | No concerns | No concerns | Very low |
| CON:RT | 4 | Some concerns | Low risk | No concerns | No concerns | Major concerns | No concerns | Low |
| AE:MBE | 0 | Some concerns | Low risk | No concerns | Major concerns | No concerns | No concerns | Low |
| CT:MBE | 0 | Major concerns | Low risk | No concerns | Major concerns | No concerns | No concerns | Very low |
| CT:RT | 0 | Some concerns | Low risk | No concerns | Major concerns | No concerns | No concerns | Low |
| MBE:RT | 0 | Some concerns | Low risk | No concerns | Major concerns | No concerns | No concerns | Low |
| **LDL-C** | | | | | | | | |
| AE:CON | 26 | Some concerns | Low risk | No concerns | No concerns | Major concerns | No concerns | Low |
| AE:CT | 4 | Major concerns | Low risk | No concerns | Some concerns | Some concerns | No concerns | Low |
| AE:RT | 4 | Some concerns | Low risk | No concerns | Major concerns | No concerns | No concerns | Low |
| CON:CT | 14 | Some concerns | Low risk | No concerns | Major concerns | No concerns | No concerns | Low |
| CON:MBE | 3 | Some concerns | Low risk | No concerns | Major concerns | No concerns | Some concerns | Low |
| CON:RT | 9 | Some concerns | Low risk | No concerns | Some concerns | Some concerns | No concerns | Low |
| CT:RT | 1 | Some concerns | Low risk | No concerns | Major concerns | No concerns | No concerns | Low |
| AE:MBE | 0 | Some concerns | Low risk | No concerns | Major concerns | No concerns | Some concerns | Low |
| CT:MBE | 0 | Some concerns | Low risk | No concerns | Major concerns | No concerns | Some concerns | Low |
| MBE:RT | 0 | Some concerns | Low risk | No concerns | Major concerns | No concerns | Some concerns | Low |
| **HDL-C** | | | | | | | | |
| AE:CON | 28 | Some concerns | Low risk | No concerns | No concerns | Major concerns | Major concerns | Very low |
| AE:CT | 4 | Some concerns | Low risk | No concerns | Some concerns | Some concerns | No concerns | Low |
| AE:RT | 3 | Some concerns | Low risk | No concerns | Major concerns | No concerns | No concerns | Low |
| CON:CT | 16 | Some concerns | Low risk | No concerns | No concerns | Major concerns | No concerns | Low |
| CON:MBE | 3 | Major concerns | Low risk | No concerns | Major concerns | No concerns | Major concerns | Low |
| CON:RT | 10 | Some concerns | Low risk | No concerns | No concerns | Major concerns | Some concerns | Low |
| CT:RT | 1 | Some concerns | Low risk | No concerns | Major concerns | No concerns | No concerns | Low |
| AE:MBE | 0 | Some concerns | Low risk | No concerns | Major concerns | No concerns | Major concerns | Very low |
| CT:MBE | 0 | Some concerns | Low risk | No concerns | Major concerns | No concerns | Major concerns | Very low |
| MBE:RT | 0 | Some concerns | Low risk | No concerns | Major concerns | No concerns | Major concerns | Very low |
| **TC** | | | | | | | | |
| AE:CON | 24 | Major concerns | Low risk | No concerns | No concerns | Some concerns | No concerns | Low |
| AE:CT | 4 | Major concerns | Low risk | No concerns | Major concerns | No concerns | No concerns | Very low |
| AE:RT | 3 | Some concerns | Low risk | No concerns | Major concerns | No concerns | No concerns | Low |
| CON:CT | 17 | Some concerns | Low risk | No concerns | No concerns | Some concerns | No concerns | Moderate |
| CON:MBE | 4 | Major concerns | Low risk | No concerns | Some concerns | Some concerns | Major concerns | Low |
| CON:RT | 10 | Some concerns | Low risk | No concerns | No concerns | Some concerns | No concerns | Moderate |
| CT:RT | 1 | Some concerns | Low risk | No concerns | Major concerns | No concerns | No concerns | Low |
| AE:MBE | 0 | Major concerns | Low risk | No concerns | No concerns | No concerns | Major concerns | Very low |
| CT:MBE | 0 | Major concerns | Low risk | No concerns | No concerns | Some concerns | Major concerns | Very low |
| MBE:RT | 0 | Some concerns | Low risk | No concerns | No concerns | Some concerns | Major concerns | Low |
| TG | | | | | | | | |
| AE:CON | 33 | Major concerns | Low risk | No concerns | No concerns | Major concerns | Some concerns | Very low |
| AE:CT | 4 | Major concerns | Low risk | No concerns | Some concerns | Some concerns | No concerns | Low |
| AE:RT | 5 | Some concerns | Low risk | No concerns | Major concerns | No concerns | No concerns | Low |
| CON:CT | 17 | Major concerns | Low risk | No concerns | No concerns | Major concerns | No concerns | Very low |
| CON:MBE | 3 | Some concerns | Low risk | No concerns | Major concerns | No concerns | Some concerns | Low |
| CON:RT | 11 | Some concerns | Low risk | No concerns | No concerns | Major concerns | No concerns | Low |
| CT:RT | 1 | Some concerns | Low risk | No concerns | Major concerns | No concerns | No concerns | Low |
| AE:MBE | 0 | Some concerns | Low risk | No concerns | Major concerns | No concerns | Some concerns | Low |
| CT:MBE | 0 | Some concerns | Low risk | No concerns | Major concerns | No concerns | Some concerns | Low |
| MBE:RT | 0 | Some concerns | Low risk | No concerns | Major concerns | No concerns | Some concerns | Low |
| **ADNP** | | | | | | | | |
| AE:CON | 5 | Some concerns | Low risk | No concerns | No concerns | No concerns | Major concerns | Low |
| AE:CT | 2 | Major concerns | Low risk | No concerns | No concerns | No concerns | Major concerns | Very low |
| CON:MBE | 1 | Some concerns | Low risk | No concerns | No concerns | No concerns | Major concerns | Low |
| CON:RT | 1 | No concerns | Low risk | No concerns | Major concerns | No concerns | Major concerns | Very low |
| AE:MBE | 0 | Some concerns | Low risk | No concerns | No concerns | No concerns | Major concerns | Low |
| AE:RT | 0 | No concerns | Low risk | No concerns | Major concerns | No concerns | Major concerns | Very low |
| CON:CT | 0 | Some concerns | Low risk | No concerns | No concerns | No concerns | Major concerns | Low |
| CT:MBE | 0 | Some concerns | Low risk | No concerns | No concerns | No concerns | Major concerns | Low |
| CT:RT | 0 | Some concerns | Low risk | No concerns | Major concerns | No concerns | Major concerns | Very low |
| MBE:RT | 0 | Some concerns | Low risk | No concerns | Major concerns | No concerns | Major concerns | Very low |
| **Leptin** | | | | | | | | |
| AE:CON | 6 | Some concerns | Low risk | No concerns | No concerns | Major concerns | Major concerns | Very low |
| AE:CT | 2 | No concerns | Low risk | No concerns | Major concerns | No concerns | Major concerns | Very low |
| CON:RT | 2 | Major concerns | Low risk | No concerns | Some concerns | Some concerns | Major concerns | Very low |
| AE:RT | 0 | Some concerns | Low risk | No concerns | Major concerns | No concerns | Major concerns | Very low |
| CON:CT | 0 | No concerns | Low risk | No concerns | Major concerns | No concerns | Major concerns | Very low |
| CT:RT | 0 | Some concerns | Low risk | No concerns | Major concerns | No concerns | Major concerns | Very low |
| ADPN: adiponectin; AE: aerobic exercise; BF: body fat; BMI: body mass index; BW: body weight; CI: confidence interval; CON: control; CT: combined training; DBP: diastolic blood pressure; Glu: glucose; HDL-C: high-density lipoprotein cholesterol; LDL-C: low-density lipoprotein cholesterol; MBE: mind-body exercise; RT: resistance training; SBP: systolic blood pressure; SMD: standardized mean difference; TC: total cholesterol; TG: triglyceride; WC: waist circumference. | | | | | | | | |

# Table S4 Pairwise meta-analysis

| **Outcome indicator** | **Comparisons** | **Number of**  **study** | **SMD** | **95%CI** | **I² (%)** | **P-value (heterogeneity)** |
| --- | --- | --- | --- | --- | --- | --- |
| BW | AE VS CON | 50 | -0.28 | -0.37, -0.20 | 48.9% | p = 0.000 |
|  | CT VS CON | 25 | -0.22 | -0.34, -0.09 | 61.1% | p = 0.001 |
|  | MBE VS CON | 9 | -0.17 | -0.39, 0.04 | 2.1% | p = 0.417 |
|  | RT VS CON | 24 | -0.19 | -0.33, -0.03 | 0.0% | p = 0.764 |
|  | OVERALL | 108 | -0.25 | -0.31, -0.19 | 42.2% | p = 0.000 |
| BF% | AE VS CON | 24 | -0.62 | -0.77, -0.47 | 81.5% | p = 0.000 |
|  | CT VS CON | 14 | -0.34 | -0.49, -0.18 | 49.6% | p = 0.018 |
|  | MBE VS CON | 3 | -0.14 | -0.54, 0.26 | 0.0% | p = 0.827 |
|  | RT VS CON | 17 | -0.58 | -0.79, -0.37 | 78.3% | p = 0.000 |
|  | OVERALL | 58 | -0.49 | -0.58, -0.39 | 75.8% | p = 0.000 |
| BMI | AE VS CON | 49 | -0.24 | -0.32, -0.16 | 63.1% | p = 0.000 |
|  | CT VS CON | 21 | -0.17 | -0.31, -0.03 | 49.9% | p = 0.005 |
|  | MBE VS CON | 9 | -0.14 | -0.39, 0.11 | 28.2% | p = 0.204 |
|  | RT VS CON | 23 | -0.23 | -0.38, -0.07 | 70.8% | p = 0.000 |
|  | OVERALL | 102 | -0.22 | -0.28, -0.16 | 60.9% | p = 0.000 |
| WC | AE VS CON | 21 | -0.30 | -0.41, -0.19 | 79.1% | p = 0.000 |
|  | CT VS CON | 8 | -0.25 | -0.47, -0.03 | 26.5% | p = 0.217 |
|  | MBE VS CON | 2 | -0.27 | -0.78, 0.26 | 62.4% | p = 0.103 |
|  | RT VS CON | 12 | -0.47 | -0.68, -0.25 | 0.0% | p = 0.947 |
|  | OVERALL | 43 | -0.32 | -0.41, -0.23 | 63.4% | p = 0.000 |
| DBP | AE VS CON | 31 | -0.21 | -0.33, -0.09 | 91.5% | p = 0.000 |
|  | CT VS CON | 11 | -0.32 | -0.47, -0.18 | 76.1% | p = 0.000 |
|  | MBE VS CON | 8 | -0.96 | -1.21, -0.71 | 84.4% | p = 0.000 |
|  | RT VS CON | 11 | -0.45 | -0.67, -0.22 | 63.3% | p = 0.002 |
|  | OVERALL | 61 | -0.35 | -0.43, -0.27 | 88.0% | p = 0.000 |
| SBP | AE VS CON | 22 | -0.45 | -0.57, -0.33 | 93.2% | p = 0.000 |
|  | CT VS CON | 11 | -0.42 | -0.57, -0.27 | 74.4% | p = 0.000 |
|  | MBE VS CON | 9 | -0.84 | -1.08, -0.59 | 91.7% | p = 0.000 |
|  | RT VS CON | 11 | -0.38 | -0.62, -0.14 | 78.8% | p = 0.000 |
|  | OVERALL | 63 | -0.48 | -0.56, -0.39 | 90.5% | p = 0.000 |
| Glu | AE VS CON | 22 | -0.22 | -0.33, -0.11 | 73.8% | p = 0.000 |
|  | CT VS CON | 10 | -0.06 | -0.25, 0.13 | 0.0% | p = 0.996 |
|  | MBE VS CON | 3 | -0.14 | -0.59, 0.32 | 80.6% | p = 0.006 |
|  | RT VS CON | 4 | -0.59 | -0.96, -0.22 | 34.3% | p = 0.206 |
|  | OVERALL | 39 | -0.20 | -0.29, -0.11 | 63.3% | p = 0.000 |
| LDL-C | AE VS CON | 26 | -0.43 | -0.56, -0.29 | 85.6% | p = 0.000 |
|  | CT VS CON | 14 | -0.19 | -0.34, -0.02 | 22.3% | p = 0.212 |
|  | MBE VS CON | 3 | 0.04 | -0.39, 0.48 | 70.3% | p = 0.035 |
|  | RT VS CON | 9 | -0.41 | -0.68, -0.13 | 46.8% | p = 0.058 |
|  | OVERALL | 52 | -0.32 | -0.41, -0.23 | 76.9% | p = 0.000 |
| HDL-C | AE VS CON | 28 | 0.36 | 0.23, 0.48 | 81.2% | p = 0.000 |
|  | CT VS CON | 16 | 0.23 | 0.08, 0.38 | 0.0% | p = 0.734 |
|  | MBE VS CON | 3 | 0.04 | -0.39, 0.47 | 0.0% | p = 0.392 |
|  | RT VS CON | 10 | 0.41 | 0.14, 0.68 | 79.5% | p = 0.000 |
|  | OVERALL | 57 | 0.30 | 0.21, 0.39 | 72.4% | p = 0.000 |
| TC | AE VS CON | 24 | -0.38 | -0.52, -0.23 | 67.0% | p = 0.000 |
|  | CT VS CON | 17 | -0.16 | -0.30, -0.03 | 69.7% | p = 0.000 |
|  | MBE VS CON | 4 | 0.17 | -0.24, 0.58 | 87.0% | p = 0.000 |
|  | RT VS CON | 10 | -0.34 | -0.59, -0.09 | 0.0% | p = 0.609 |
|  | OVERALL | 55 | -0.25 | -0.34, -0.16 | 66.6% | p = 0.000 |
| TG | AE VS CON | 33 | -0.19 | -0.29, -0.08 | 85.6% | p = 0.000 |
|  | CT VS CON | 17 | -0.18 | -0.31, -0.04 | 43.8% | p = 0.028 |
|  | MBE VS CON | 3 | -0.35 | -0.87, 0.17 | 0.0% | p = 0.727 |
|  | RT VS CON | 11 | -0.22 | -0.47, 0.03 | 47.4% | p = 0.040 |
|  | OVERALL | 64 | -0.19 | -0.27, -0.11 | 76.8% | p = 0.000 |
| ADPN | AE VS CON | 5 | 0.08 | -0.06, 0.22 | 49.4% | p = 0.095 |
|  | MBE VS CON | 1 | 1.84 | 0.64, 3.03 | NA | NA |
|  | RT VS CON | 1 | 0.00 | -0.54, 0.54 | NA | NA |
|  | OVERALL | 7 | 0.10 | -0.04, 0.23 | 63.1% | p = 0.012 |
| Leptin | AE VS CON | 6 | -0.30 | -0.44, -0.16 | 52.4% | p = 0.062 |
|  | RT VS CON | 2 | -0.48 | -0.99, 0.03 | 96.9% | p = 0.000 |
|  | OVERALL | 8 | -0.31 | -0.45, -0.18 | 83.9% | p = 0.000 |
| ADPN: adiponectin; AE: aerobic exercise; BF: body fat; BMI: body mass index; BW: body weight; CI: confidence interval; CON: control; CT: combined training; DBP: diastolic blood pressure; Glu: glucose; HDL-C: high-density lipoprotein cholesterol; LDL-C: low-density lipoprotein cholesterol; MBE: mind-body exercise; RT: resistance training; SBP: systolic blood pressure; SMD: standardized mean difference; TC: total cholesterol; TG: triglyceride; WC: waist circumference. | | | | | | |

# Table S5 Matrix of the network meta-analysis results for body composition (BW, BF%, BMI and WC).

| BW | AE |  |  |  |  |
| --- | --- | --- | --- | --- | --- |
|  | -1.43 (-1.97, -0.9) * | Control |  |  |  |
|  | 0.41 (-0.57, 1.37) | 1.84 (0.95, 2.71) * | CT |  |  |
|  | -0.66 (-2.33, 1.05) | 0.77 (-0.84, 2.4) | -1.07 (-2.87, 0.78) | MBE |  |
|  | -0.54 (-1.61, 0.55) | 0.89 (-0.11, 1.91) | -0.95 (-2.24, 0.38) | 0.12 (-1.76, 1.99) | RT |
| BF% | AE |  |  |  |  |
|  | -2.22 (-3.15, -1.28) * | Control |  |  |  |
|  | -0.5 (-1.98, 0.97) | 1.71 (0.46, 2.96) * | CT |  |  |
|  | -1.69 (-4, 0.65) | 0.52 (-1.63, 2.71) | -1.19 (-3.67, 1.33) | MBE |  |
|  | -0.45 (-1.77, 0.89) | 1.77 (0.7, 2.85) * | 0.05 (-1.55, 1.67) | 1.24 (-1.08, 3.53) | RT |
| BMI | AE |  |  |  |  |
|  | -0.52 (-0.78, -0.26) * | Control |  |  |  |
|  | 0.31 (-0.2, 0.81) | 0.83 (0.35, 1.3) * | CT |  |  |
|  | -0.24 (-0.88, 0.41) | 0.28 (-0.32, 0.88) | -0.55 (-1.31, 0.22) | MBE |  |
|  | -0.08 (-0.57, 0.41) | 0.44 (0, 0.88) * | -0.39 (-1.03, 0.25) | 0.16 (-0.57, 0.88) | RT |
| WC | AE |  |  |  |  |
|  | -2.25 (-3.23, -1.29) * | Control |  |  |  |
|  | -0.48 (-2.46, 1.51) | 1.77 (0.02, 3.56) * | CT |  |  |
|  | -0.71 (-3.6, 2.13) | 1.54 (-1.24, 4.28) | -0.23 (-3.55, 3.01) | MBE |  |
|  | 0.55 (-1.15, 2.29) | 2.8 (1.28, 4.36) * | 1.03 (-1.3, 3.37) | 1.26 (-1.83, 4.45) | RT |
| AE: aerobic exercise; BF: body fat; BMI: body mass index; BW: body weight; CT: combined training; MBE: mind-body exercise; RT: resistance training; WC: waist circumference. | | | | | |

# Table S6 Matrix of the network meta-analysis results for blood pressure (DBP, SBP).

| DBP | AE |  |  |  |  |
| --- | --- | --- | --- | --- | --- |
|  | -2.5 (-3.8, -1.18) * | Control |  |  |  |
|  | -0.29 (-2.68, 2.13) | 2.21 (0.02, 4.41) * | CT |  |  |
|  | 2.97 (0.27, 5.75) * | 5.47 (3.02, 7.99) * | 3.26 (0.06, 6.51) * | MBE |  |
|  | 0.9 (-1.38, 3.24) | 3.4 (1.3, 5.55) * | 1.19 (-1.79, 4.21) | -2.07 (-5.08, 0.94) | RT |
| SBP | AE |  |  |  |  |
|  | -5.11 (-7.02, -3.17) * | Control |  |  |  |
|  | -1.64 (-5.27, 2.04) | 3.47 (0.18, 6.74) * | CT |  |  |
|  | 2.1 (-1.75, 5.95) | 7.21 (3.74, 10.63) * | 3.74 (-0.85, 8.3) | MBE |  |
|  | -0.53 (-3.87, 2.91) | 4.58 (1.46, 7.74) * | 1.11 (-3.34, 5.64) | -2.64 (-6.87, 1.7) | RT |
| AE: aerobic exercise; CT: combined training; DBP: diastolic blood pressure; MBE: mind-body exercise; RT: resistance training; SBP: systolic blood pressure. | | | | | |

# Table S7 Matrix of the network meta-analysis results for Glu.

| Glu | AE |  |  |  |  |
| --- | --- | --- | --- | --- | --- |
|  | -3.66 (-6.32, -1.03) * | Control |  |  |  |
|  | -2.49 (-6.77, 1.86) | 1.16 (-2.71, 5.1) | CT |  |  |
|  | -0.91 (-9.26, 7.33) | 2.74 (-5.15, 10.59) | 1.59 (-7.3, 10.28) | MBE |  |
|  | 4.57 (-2.42, 11.72) | 8.24 (1.52, 15.1) * | 7.07 (-0.68, 14.89) | 5.48 (-4.83, 15.99) | RT |
| AE: aerobic exercise; CT: combined training; Glu: glucose; MBE: mind-body exercise; RT: resistance training. | | | | | |

# Table S8 Matrix of the network meta-analysis results for Cholesterol and Blood lipid (LDL-C, HDL-C, TC and TG).

| LDL-C | AE |  |  |  |  |
| --- | --- | --- | --- | --- | --- |
|  | -8.36 (-13.11, -3.9) * | Control |  |  |  |
|  | -5.48 (-13.52, 2.34) | 2.9 (-4.07, 9.8) | CT |  |  |
|  | -2.95 (-19.18, 13.79) | 5.42 (-10.14, 21.75) | 2.56 (-14.49, 20.32) | MBE |  |
|  | -0.11 (-9.69, 9.45) | 8.26 (-0.68, 17.46) | 5.38 (-5.78, 16.77) | 2.83 (-15.69, 20.82) | RT |
| HDL-C | AE |  |  |  |  |
|  | 3.23 (1.53, 4.92) * | Control |  |  |  |
|  | 1.02 (-1.81, 3.78) | -2.21 (-4.6, 0.11) | CT |  |  |
|  | 1.99 (-4.13, 7.95) | -1.24 (-7.09, 4.45) | 0.98 (-5.28, 7.14) | MBE |  |
|  | 1 (-2, 4.01) | -2.23 (-5.01, 0.56) | -0.02 (-3.56, 3.61) | -0.99 (-7.32, 5.5) | RT |
| TC | AE |  |  |  |  |
|  | -6.26 (-10.56, -2.18) * | Control |  |  |  |
|  | -2.21 (-8.95, 4.34) | 4.06 (-1.5, 9.58) | CT |  |  |
|  | -2.38 (-15.27, 11.51) | 3.9 (-8.32, 17.21) | -0.16 (-13.5, 14.33) | MBE |  |
|  | 1.42 (-6.48, 9.61) | 7.69 (0.5, 15.32) * | 3.64 (-5.35, 13.02) | 3.81 (-11.14, 17.97) | RT |
| TG | AE |  |  |  |  |
|  | -5.62 (-10.68, -0.54) * | Control |  |  |  |
|  | 5.86 (-3.12, 15.08) | 11.49 (3.65, 19.53) * | CT |  |  |
|  | 6.81 (-18.51, 32.51) | 12.45 (-12.46, 37.58) | 0.96 (-25.18, 27.27) | MBE |  |
|  | 1.22 (-8.81, 11.26) | 6.83 (-2.42, 16.12) | -4.64 (-16.82, 7.29) | -5.62 (-32.35, 21.08) | RT |
| AE: aerobic exercise; CT: combined training; MBE: mind-body exercise; RT: resistance training; HDL-C: high-density lipoprotein cholesterol; LDL-C: low-density lipoprotein cholesterol; TC: total cholesterol; TG: triglyceride. | | | | | |

# Table S9 Matrix of the network meta-analysis results for ADPN and Leptin.

| ADPN | AE |  |  |  |  |
| --- | --- | --- | --- | --- | --- |
|  | 0.11 (-0.39, 0.73) | Control |  |  |  |
|  | -1.27 (-2.72, 0.2) | -1.39 (-2.94, 0.14) | CT |  |  |
|  | -1.84 (-3.31, -0.32) * | -1.96 (-3.37, -0.58) * | -0.58 (-2.66, 1.51) | MBE |  |
|  | 0.67 (-21.43, 19.82) | 0.54 (-21.55, 19.68) | 2.02 (-20.16, 21.19) | 2.54 (-19.55, 21.67) | RT |
| Leptin | AE |  |  |  |  |
|  | -1.94 (-4.38, 0.45) | Control |  |  |  |
|  | 0.59 (-6.45, 6.8) | 2.53 (-4.96, 9.18) | CT |  |  |
|  | 0.64 (-4.84, 5.85) | 2.56 (-2.29, 7.25) | 0.02 (-8.01, 8.86) | RT |  |
| AE: aerobic exercise; CT: combined training; MBE: mind-body exercise; RT: resistance training; ADPN: adiponectin. | | | | |  |


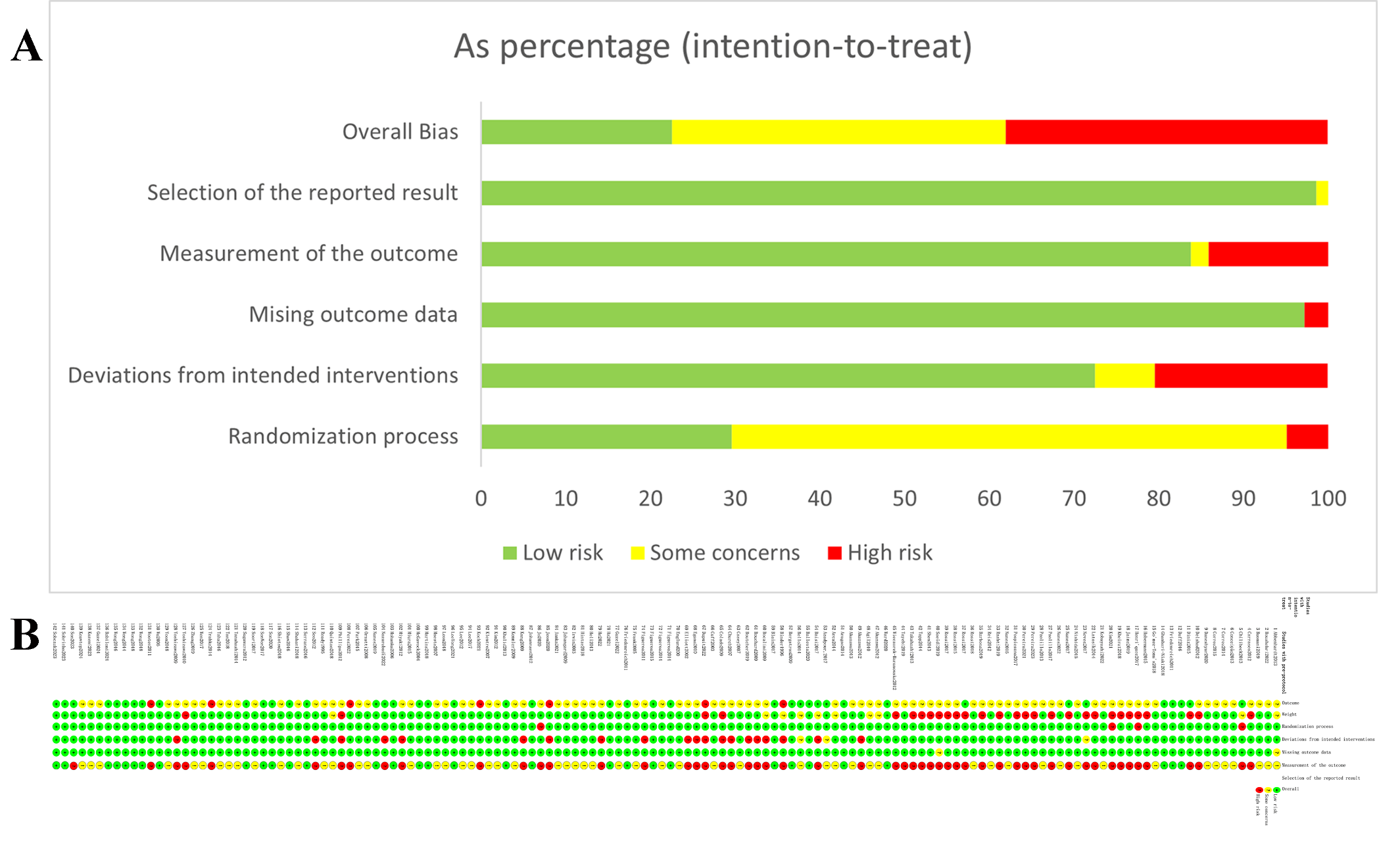


**Supplementary Figure 1** Risk of bias graph. A. The risk of bias summary: review authors’ judgements about each risk of bias item for each included study; B. The risk of bias graph: review authors’ judgements about each risk of bias item presented as percentages across all included 142 studies.


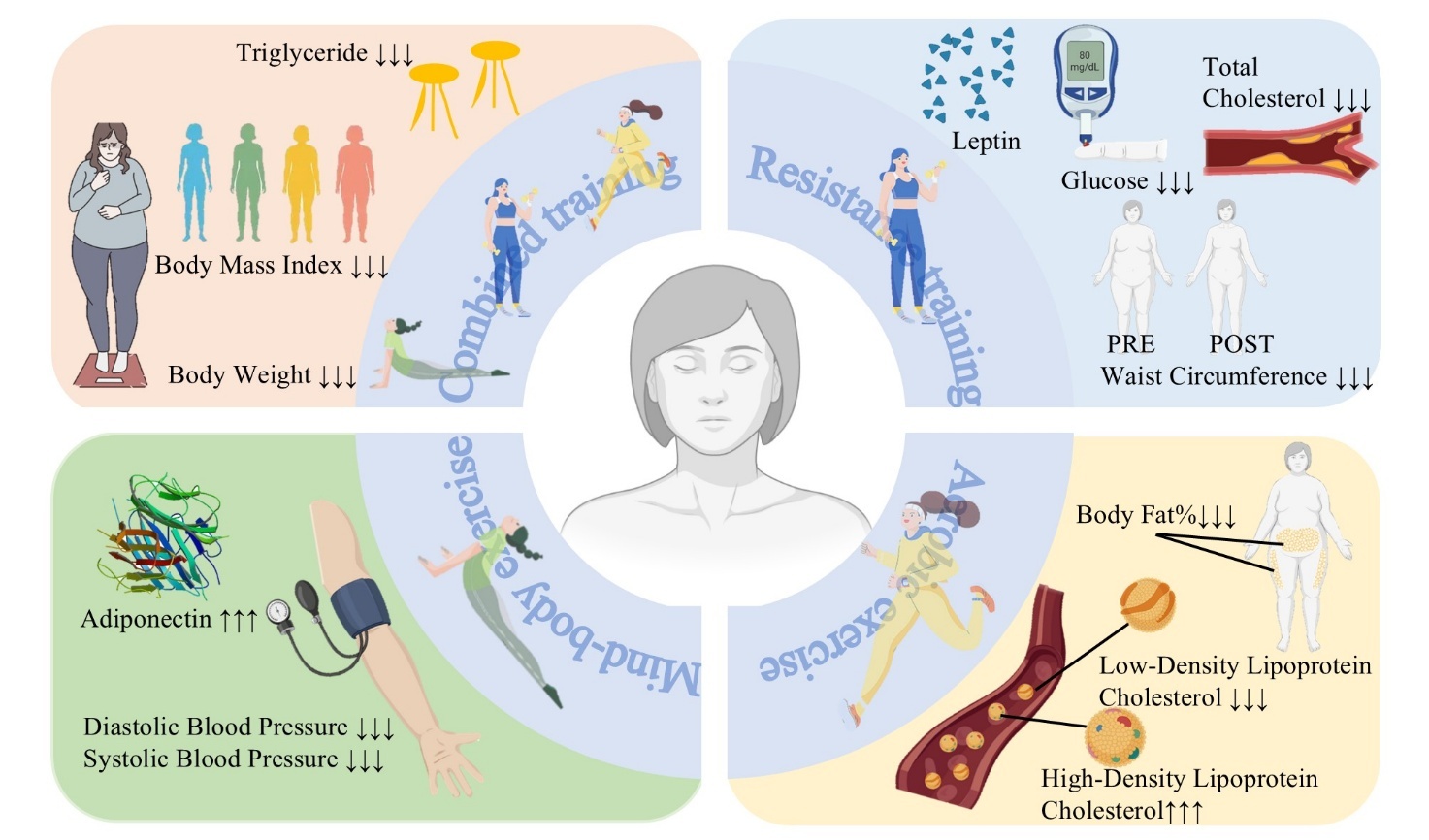


**Supplementary Figure 2** The optimal exercise interventions for metabolic syndrome risk factors in postmenopausal women.

**References**

[1] Abbenhardt C, McTiernan A, Alfano CM, et al. Effects of individual and combined dietary weight loss and exercise interventions in postmenopausal women on adiponectin and leptin levels. J Intern Med. 2013;274(2):163-175. doi:10.1111/joim.12062.

[2] Ağıl A, Abıke F, Daşkapan A, et al. Short-term exercise approaches on menopausal symptoms, psychological health, and quality of life in postmenopausal women. Obstet Gynecol Int. 2010;2010doi:10.1155/2010/274261.

[3] Akazawa N, Choi Y, Miyaki A, et al. Aerobic exercise training increases cerebral blood flow in postmenopausal women. Artery Research. 2012;6(3):124-129. doi:10.1016/j.artres.2012.05.003.

[4] Akazawa N, Choi Y, Miyaki A, et al. Curcumin ingestion and exercise training improve vascular endothelial function in postmenopausal women. Nutr Res. 2012;32(10):795-799. doi:10.1016/j.nutres.2012.09.002.

[5] Akazawa N, Choi Y, Miyaki A, et al. Effects of curcumin intake and aerobic exercise training on arterial compliance in postmenopausal women. Artery Research. 2013;7(1):67-72. doi:10.1016/j.artres.2012.09.003.

[6] Aragão FR, Abrantes CG, Gabriel RE, et al. Effects of a 12-month multi-component exercise program on the body composition of postmenopausal women. Climacteric. 2014;17(2):155-163. doi:10.3109/13697137.2013.819328.

[7] Arca EA, Martinelli B, Martin LC, et al. Aquatic exercise is as effective as dry land training to blood pressure reduction in postmenopausal hypertensive women. Physiother Res Int. 2014;19(2):93-98. doi:10.1002/pri.1565.

[8] Azadpour N, Tartibian B, Koşar Ş N. Effects of aerobic exercise training on ACE and ADRB2 gene expression, plasma angiotensin II level, and flow-mediated dilation: a study on obese postmenopausal women with prehypertension. Menopause. 2017;24(3):269-277. doi:10.1097/gme.0000000000000762.

[9] Babiloni-Lopez C, Gargallo P, Juesas A, et al. Long-Term Effects of Microfiltered Seawater and Resistance Training with Elastic Bands on Hepatic Parameters, Inflammation, Oxidative Stress, and Blood Pressure of Older Women: A 32-Week, Double-Blinded, Randomized, Placebo-Controlled Trial. Healthcare (Basel). 2024;12(2)doi:10.3390/healthcare12020204.

[10] Chagas EFB, Bonfim MR, Turi BC, et al. Effect of Moderate-Intensity Exercise on Inflammatory Markers Among Postmenopausal Women. J Phys Act Health. 2017;14(6):479-485. doi:10.1123/jpah.2016-0319.

[11] Ballesta-García I, Martínez-González-Moro I, Ramos-Campo DJ, et al. High-Intensity Interval Circuit Training Versus Moderate-Intensity Continuous Training on Cardiorespiratory Fitness in Middle-Aged and Older Women: A Randomized Controlled Trial. Int J Environ Res Public Health. 2020;17(5)doi:10.3390/ijerph17051805.

[12] Rezende Barbosa MP, Vanderlei LC, Neves LM, et al. Functional training in postmenopause: Cardiac autonomic modulation and cardiorespiratory parameters, a randomized trial. Geriatr Gerontol Int. 2019;19(8):823-828. doi:10.1111/ggi.13690.

[13] Bello M, Sousa MC, Neto G, et al. The effect of a long-term, community-based exercise program on bone mineral density in postmenopausal women with pre-diabetes and type 2 diabetes. J Hum Kinet. 2014;43:43-48. doi:10.2478/hukin-2014-0088.

[14] Bergström I, Lombardo C, Brinck J. Physical training decreases waist circumference in postmenopausal borderline overweight women. Acta Obstet Gynecol Scand. 2009;88(3):308-313. doi:10.1080/00016340802695942.

[15] Binder EF, Birge SJ, Kohrt WM. Effects of endurance exercise and hormone replacement therapy on serum lipids in older women. J Am Geriatr Soc. 1996;44(3):231-236. doi:10.1111/j.1532-5415.1996.tb00907.x.

[16] Blain H, Jaussent A, Picot MC, et al. Effect of a 6-Month Brisk Walking Program on Walking Endurance in Sedentary and Physically Deconditioned Women Aged 60 or Older: A Randomized Trial. J Nutr Health Aging. 2017;21(10):1183-1189. doi:10.1007/s12603-017-0955-7.

[17] Bocalini DS, Serra AJ, dos Santos L, et al. Strength training preserves the bone mineral density of postmenopausal women without hormone replacement therapy. J Aging Health. 2009;21(3):519-527. doi:10.1177/0898264309332839.

[18] Bouchard DR, Soucy L, Sénéchal M, et al. Impact of resistance training with or without caloric restriction on physical capacity in obese older women. Menopause. 2009;16(1):66-72. doi:10.1097/gme.0b013e31817dacf7.

[19] Boushehri SN, Farazmand M, Zar A. Dietary assessment and effect of Pilates exercises on quality of life, body composition, and physical fitness in Iranian postmenopausal women. Comparative Exercise Physiology. 2022;18(3):249-254.

[20] Boutcher YN, Boutcher SH, Yoo HY, et al. The Effect of Sprint Interval Training on Body Composition of Postmenopausal Women. Med Sci Sports Exerc. 2019;51(7):1413-1419. doi:10.1249/mss.0000000000001919.

[21] Buonani C, Rossi FE, Diniz TA, et al. Concurrent training and taurine improve lipid profile in postmenopausal women. Revista Brasileira de Medicina do Esporte. 2019;25:121-126.

[22] Carrasco M, Vaquero M. Water training in postmenopausal women: Effect on muscular strength. European Journal of Sport Science. 2012;12(2):193-200.

[23] Casey DP, Pierce GL, Howe KS, et al. Effect of resistance training on arterial wave reflection and brachial artery reactivity in normotensive postmenopausal women. Eur J Appl Physiol. 2007;100(4):403-408. doi:10.1007/s00421-007-0447-2.

[24] Chilibeck PD, Vatanparast H, Pierson R, et al. Effect of exercise training combined with isoflavone supplementation on bone and lipids in postmenopausal women: a randomized clinical trial. J Bone Miner Res. 2013;28(4):780-793. doi:10.1002/jbmr.1815.

[25] Church TS, Earnest CP, Skinner JS, et al. Effects of different doses of physical activity on cardiorespiratory fitness among sedentary, overweight or obese postmenopausal women with elevated blood pressure: a randomized controlled trial. Jama. 2007;297(19):2081-2091. doi:10.1001/jama.297.19.2081.

[26] Colado JC, Triplett NT, Tella V, et al. Effects of aquatic resistance training on health and fitness in postmenopausal women. Eur J Appl Physiol. 2009;106(1):113-122. doi:10.1007/s00421-009-0996-7.

[27] Conceição MS, Bonganha V, Vechin FC, et al. Sixteen weeks of resistance training can decrease the risk of metabolic syndrome in healthy postmenopausal women. Clin Interv Aging. 2013;8:1221-1228. doi:10.2147/cia.S44245.

[28] Correa CS, Teixeira BC, Bittencourt A, et al. Effects of high and low volume of strength training on muscle strength, muscle volume and lipid profile in postmenopausal women. Journal of Exercise Science & Fitness. 2014;12(2):62-67.

[29] Correa CS, Teixeira BC, Cobos RC, et al. High-volume resistance training reduces postprandial lipaemia in postmenopausal women. J Sports Sci. 2015;33(18):1890-1901. doi:10.1080/02640414.2015.1017732.

[30] Cuff DJ, Meneilly GS, Martin A, et al. Effective exercise modality to reduce insulin resistance in women with type 2 diabetes. Diabetes Care. 2003;26(11):2977-2982. doi:10.2337/diacare.26.11.2977.

[31] Daneshyar F, Moradi F, Atashak S. Effect of yoga with and without elastic band resistance training on visfatin, liver enzymes and body composition in postmenopausal women. Comparative Exercise Physiology. 2020;16(5):347-355.

[32] Delshad M, Ghanbarian A, Mehrabi Y, et al. Effect of Strength Training and Short-term Detraining on Muscle Mass in Women Aged Over 50 Years Old. Int J Prev Med. 2013;4(12):1386-1394.

[33] Diniz T, Fortaleza A, Rossi F, et al. Short-term program of aerobic training prescribed using critical velocity is effective to improve metabolic profile in postmenopausal women. Science & Sports. 2016;31(2):95-102.

[34] Duff WR, Kontulainen SA, Candow DG, et al. Effects of low-dose ibuprofen supplementation and resistance training on bone and muscle in postmenopausal women: A randomized controlled trial. Bone Rep. 2016;5:96-103. doi:10.1016/j.bonr.2016.04.004.

[35] Dupuit M, Rance M, Morel C, et al. Effect of Concurrent Training on Body Composition and Gut Microbiota in Postmenopausal Women with Overweight or Obesity. Med Sci Sports Exerc. 2022;54(3):517-529. doi:10.1249/mss.0000000000002809.

[36] Egaña M, Reilly H, Green S. Effect of elastic-band-based resistance training on leg blood flow in elderly women. Appl Physiol Nutr Metab. 2010;35(6):763-772. doi:10.1139/h10-071.

[37] Elliott KJ, Sale C, Cable NT. Effects of resistance training and detraining on muscle strength and blood lipid profiles in postmenopausal women. Br J Sports Med. 2002;36(5):340-344. doi:10.1136/bjsm.36.5.340.

[38] Englund U, Littbrand H, Sondell A, et al. A 1-year combined weight-bearing training program is beneficial for bone mineral density and neuromuscular function in older women. Osteoporos Int. 2005;16(9):1117-1123. doi:10.1007/s00198-004-1821-0.

[39] Figueroa A, Park SY, Seo DY, et al. Combined resistance and endurance exercise training improves arterial stiffness, blood pressure, and muscle strength in postmenopausal women. Menopause. 2011;18(9):980-984. doi:10.1097/gme.0b013e3182135442.

[40] Figueroa A, Kalfon R, Madzima TA, et al. Effects of whole-body vibration exercise training on aortic wave reflection and muscle strength in postmenopausal women with prehypertension and hypertension. J Hum Hypertens. 2014;28(2):118-122. doi:10.1038/jhh.2013.59.

[41] Figueroa A, Kalfon R, Madzima TA, et al. Whole-body vibration exercise training reduces arterial stiffness in postmenopausal women with prehypertension and hypertension. Menopause. 2014;21(2):131-136. doi:10.1097/GME.0b013e318294528c.

[42] Figueroa A, Kalfon R, Wong A. Whole-body vibration training decreases ankle systolic blood pressure and leg arterial stiffness in obese postmenopausal women with high blood pressure. Menopause. 2015;22(4):423-427. doi:10.1097/gme.0000000000000332.

[43] Frank LL, Sorensen BE, Yasui Y, et al. Effects of exercise on metabolic risk variables in overweight postmenopausal women: a randomized clinical trial. Obes Res. 2005;13(3):615-625. doi:10.1038/oby.2005.66.

[44] Friedenreich CM, Neilson HK, Woolcott CG, et al. Changes in insulin resistance indicators, IGFs, and adipokines in a year-long trial of aerobic exercise in postmenopausal women. Endocr Relat Cancer. 2011;18(3):357-369. doi:10.1530/erc-10-0303.

[45] Friedenreich CM, Woolcott CG, McTiernan A, et al. Adiposity changes after a 1-year aerobic exercise intervention among postmenopausal women: a randomized controlled trial. Int J Obes (Lond). 2011;35(3):427-435. doi:10.1038/ijo.2010.147.

[46] Gerage AM, Forjaz CL, Nascimento MA, et al. Cardiovascular adaptations to resistance training in elderly postmenopausal women. Int J Sports Med. 2013;34(9):806-813. doi:10.1055/s-0032-1331185.

[47] Ghanbari-Niaki A, Saeidi A, Ahmadian M, et al. The combination of exercise training and Zataria multiflora supplementation increase serum irisin levels in postmenopausal women. Integr Med Res. 2018;7(1):44-52. doi:10.1016/j.imr.2018.01.007.

[48] Gómez-Tomás C, Chulvi-Medrano I, Carrasco JJ, et al. Effect of a 1-year elastic band resistance exercise program on cardiovascular risk profile in postmenopausal women. Menopause. 2018;25(9):1004-1010. doi:10.1097/gme.0000000000001113.

[49] Guzel Y, Atakan MM, Areta JL, et al. Ten weeks of low-volume walking training improve cardiometabolic health and body composition in sedentary postmenopausal women with obesity without affecting markers of bone metabolism. Res Sports Med. 2024;32(2):331-343. doi:10.1080/15438627.2022.2113877.

[50] Ha MS, Yook JS, Lee M, et al. Exercise training and burdock root (Arctium lappa L.) extract independently improve abdominal obesity and sex hormones in elderly women with metabolic syndrome. Sci Rep. 2021;11(1):5175. doi:10.1038/s41598-021-84301-x.

[51] Habermann N, Makar KW, Abbenhardt C, et al. No effect of caloric restriction or exercise on radiation repair capacity. Med Sci Sports Exerc. 2015;47(5):896-904. doi:10.1249/mss.0000000000000480.

[52] He H, Wang C, Chen X, et al. The effects of HIIT compared to MICT on endothelial function and hemodynamics in postmenopausal females. J Sci Med Sport. 2022;25(5):364-371. doi:10.1016/j.jsams.2022.01.007.

[53] Heli V, Ihab H, Kun H, et al. Effects of exercise program on physiological functions in postmenopausal women with metabolic syndrome. Int J Gerontol. 2013;7(4):231-235. doi:10.1016/j.ijge.2013.05.002.

[54] Henríquez S, Monsalves-Alvarez M, Jimenez T, et al. Effects of Two Training Modalities on Body Fat and Insulin Resistance in Postmenopausal Women. J Strength Cond Res. 2017;31(11):2955-2964. doi:10.1519/jsc.0000000000002089.

[55] Hintze LJ, Messier V, Lavoie M, et al. A one-year resistance training program following weight loss has no significant impact on body composition and energy expenditure in postmenopausal women living with overweight and obesity. Physiol Behav. 2018;189:99-106. doi:10.1016/j.physbeh.2018.03.014.

[56] Irwin ML, Yasui Y, Ulrich CM, et al. Effect of exercise on total and intra-abdominal body fat in postmenopausal women: a randomized controlled trial. Jama. 2003;289(3):323-330. doi:10.1001/jama.289.3.323.

[57] Jahangard T, Torkaman G, Ghoosheh B, et al. The effect of short-term aerobic training on coagulation and fibrinolytic factors in sedentary healthy postmenopausal women. Maturitas. 2009;64(4):223-227. doi:10.1016/j.maturitas.2009.09.003.

[58] Jaime SJ, Maharaj A, Alvarez-Alvarado S, et al. Impact of low-intensity resistance and whole-body vibration training on aortic hemodynamics and vascular function in postmenopausal women. Hypertens Res. 2019;42(12):1979-1988. doi:10.1038/s41440-019-0328-1.

[59] Jamka M, Mądry E, Bogdański P, et al. The Effect of Endurance and Endurance-Strength Training on Bone Mineral Density and Content in Abdominally Obese Postmenopausal Women: A Randomized Trial. Healthcare (Basel). 2021;9(8)doi:10.3390/healthcare9081074.

[60] Jeon YK, Kim SS, Kim JH, et al. Combined Aerobic and Resistance Exercise Training Reduces Circulating Apolipoprotein J Levels and Improves Insulin Resistance in Postmenopausal Diabetic Women. Diabetes Metab J. 2020;44(1):103-112. doi:10.4093/dmj.2018.0160.

[61] Jo EA, Wu SS, Han HR, et al. Effects of exergaming in postmenopausal women with high cardiovascular risk: A randomized controlled trial. Clin Cardiol. 2020;43(4):363-370. doi:10.1002/clc.23324.

[62] Johannsen NM, Swift DL, Johnson WD, et al. Effect of different doses of aerobic exercise on total white blood cell (WBC) and WBC subfraction number in postmenopausal women: results from DREW. PLoS One. 2012;7(2):e31319. doi:10.1371/journal.pone.0031319.

[63] Kang S, Woo JH, Shin KO, et al. Circuit resistance exercise improves glycemic control and adipokines in females with type 2 diabetes mellitus. J Sports Sci Med. 2009;8(4):682-688.

[64] Kazemi SS, Heidarianpour A, Shokri E. Effect of resistance training and high-intensity interval training on metabolic parameters and serum level of Sirtuin1 in postmenopausal women with metabolic syndrome: a randomized controlled trial. Lipids Health Dis. 2023;22(1):177. doi:10.1186/s12944-023-01940-x.

[65] Keawtep P, Sungkarat S, Boripuntakul S, et al. Effects of combined dietary intervention and physical-cognitive exercise on cognitive function and cardiometabolic health of postmenopausal women with obesity: a randomized controlled trial. Int J Behav Nutr Phys Act. 2024;21(1):28. doi:10.1186/s12966-024-01580-z.

[66] Kemmler W, Von Stengel S, Engelke K, et al. Exercise decreases the risk of metabolic syndrome in elderly females. Med Sci Sports Exerc. 2009;41(2):297-305. doi:10.1249/MSS.0b013e31818844b7.

[67] Khalid T, Nesreen E, Ramadhan O. Effects of exercise training on postmenopausal hypertension: implications on nitric oxide levels. Med J Malaysia. 2013;68(6):459-464.

[68] Kheirat F, Merzouk H, Merzouk AS, et al. One year changes in biochemical and redox markers in training menopausal women with adherence to Mediterranean diet. Science & Sports. 2018;33(1):e25-e32.

[69] Kim JW, Kim DY. Effects of aerobic exercise training on serum sex hormone binding globulin, body fat index, and metabolic syndrome factors in obese postmenopausal women. Metab Syndr Relat Disord. 2012;10(6):452-457. doi:10.1089/met.2012.0036.

[70] Kim JH, Ha MS, Ha SM, et al. Aquatic Exercise Positively Affects Physiological Frailty among Postmenopausal Women: A Randomized Controlled Clinical Trial. Healthcare (Basel). 2021;9(4)doi:10.3390/healthcare9040409.

[71] Klentrou P, Slack J, Roy B, et al. Effects of exercise training with weighted vests on bone turnover and isokinetic strength in postmenopausal women. J Aging Phys Act. 2007;15(3):287-299. doi:10.1123/japa.15.3.287.

[72] Kobayashi R, Asaki K, Hashiguchi T, et al. Effect of aerobic exercise training frequency on arterial stiffness in middle-aged and elderly females. J Phys Ther Sci. 2022;34(5):347-352. doi:10.1589/jpts.34.347.

[73] Buttelli ACK, Costa RR, Farinha JB, et al. Pilates training improves aerobic capacity, but not lipid or lipoprotein levels in elderly women with dyslipidemia: A controlled trial. J Bodyw Mov Ther. 2021;26:227-232. doi:10.1016/j.jbmt.2020.10.007.

[74] Latosik E, Zubrzycki IZ, Ossowski Z, et al. Physiological Responses Associated with Nordic-walking training in Systolic Hypertensive Postmenopausal Women. J Hum Kinet. 2014;43:185-190. doi:10.2478/hukin-2014-0104.

[75] Lee JA, Kim JW, Kim DY. Effects of yoga exercise on serum adiponectin and metabolic syndrome factors in obese postmenopausal women. Menopause. 2012;19(3):296-301. doi:10.1097/gme.0b013e31822d59a2.

[76] Lee J-A, Kim J-H, Kim J-W, et al. Effects of Aerobic Exercise on Serum Blood Lipids, Leptin, Ghrelin, and HOMA-IR Factors in Postmenopausal Obese Women. Journal of the Korea Academia-Industrial cooperation Society. 2017;18(2):549-558. doi:10.5762/KAIS.2017.18.2.549.

[77] Lee YK, Cho SY, Roh HT. Effects of 16 Weeks of Taekwondo Training on the Cerebral Blood Flow Velocity, Circulating Neurotransmitters, and Subjective Well-Being of Obese Postmenopausal Women. Int J Environ Res Public Health. 2021;18(20)doi:10.3390/ijerph182010789.

[78] Jeon K, Lee S, Hwang MH. Effect of combined circuit exercise on arterial stiffness in hypertensive postmenopausal women: a local public health center-based pilot study. Menopause. 2018;25(12):1442-1447. doi:10.1097/gme.0000000000001154.

[79] Maesta N, Nahas EA, Nahas-Neto J, et al. Effects of soy protein and resistance exercise on body composition and blood lipids in postmenopausal women. Maturitas. 2007;56(4):350-358. doi:10.1016/j.maturitas.2006.10.001.

[80] Martins FM, de Paula Souza A, Nunes PRP, et al. High-intensity body weight training is comparable to combined training in changes in muscle mass, physical performance, inflammatory markers and metabolic health in postmenopausal women at high risk for type 2 diabetes mellitus: A randomized controlled clinical trial. Exp Gerontol. 2018;107:108-115. doi:10.1016/j.exger.2018.02.016.

[81] McGavock J, Mandic S, Lewanczuk R, et al. Cardiovascular adaptations to exercise training in postmenopausal women with type 2 diabetes mellitus. Cardiovasc Diabetol. 2004;3:3. doi:10.1186/1475-2840-3-3.

[82] Miura H, Takahashi Y, Maki Y, et al. Effects of exercise training on arterial stiffness in older hypertensive females. Eur J Appl Physiol. 2015;115(9):1847-1854. doi:10.1007/s00421-015-3168-y.

[83] Miyaki A, Maeda S, Choi Y, et al. Habitual aerobic exercise increases plasma pentraxin 3 levels in middle-aged and elderly women. Appl Physiol Nutr Metab. 2012;37(5):907-911. doi:10.1139/h2012-069.

[84] Mohanka M, Irwin M, Heckbert SR, et al. Serum lipoproteins in overweight/obese postmenopausal women: a one-year exercise trial. Med Sci Sports Exerc. 2006;38(2):231-239. doi:10.1249/01.mss.0000184584.95000.e4.

[85] Nazarabadi PN, Etemad Z, Hoseini R, et al. Anti-Inflammatory Effects of a Period of Aerobic Training and Vitamin D Supplementation in Postmenopausal Women with Metabolic Syndrome. Int J Prev Med. 2022;13:60. doi:10.4103/ijpvm.IJPVM_312_20.

[86] Neves LM, Fortaleza AC, Rossi FE, et al. Functional training reduces body fat and improves functional fitness and cholesterol levels in postmenopausal women: a randomized clinical trial. J Sports Med Phys Fitness. 2017;57(4):448-456. doi:10.23736/s0022-4707.17.06062-5.

[87] Nishida Y, Tanaka K, Hara M, et al. Effects of home-based bench step exercise on inflammatory cytokines and lipid profiles in elderly Japanese females: A randomized controlled trial. Arch Gerontol Geriatr. 2015;61(3):443-451. doi:10.1016/j.archger.2015.06.017.

[88] Nunes PR, Barcelos LC, Oliveira AA, et al. Effect of resistance training on muscular strength and indicators of abdominal adiposity, metabolic risk, and inflammation in postmenopausal women: controlled and randomized clinical trial of efficacy of training volume. Age (Dordr). 2016;38(2):40. doi:10.1007/s11357-016-9901-6.

[89] Nunes PRP, Oliveira AA, Martins FM, et al. Effect of resistance training volume on walking speed performance in postmenopausal women: A randomized controlled trial. Exp Gerontol. 2017;97:80-88. doi:10.1016/j.exger.2017.08.011.

[90] Nunes PRP, Barcelos LC, Oliveira AA, et al. Muscular Strength Adaptations and Hormonal Responses After Two Different Multiple-Set Protocols of Resistance Training in Postmenopausal Women. J Strength Cond Res. 2019;33(5):1276-1285. doi:10.1519/jsc.0000000000001788.

[91] Nunes PRP, Silva T, Carneiro MAS, et al. Functional high-intensity interval training is not equivalent when compared to combined training for blood pressure improvements in postmenopausal women: a randomized controlled trial. Clin Exp Hypertens. 2022;44(2):127-133. doi:10.1080/10641963.2021.2001481.

[92] Orsatti FL, Nahas EA, Maesta N, et al. Plasma hormones, muscle mass and strength in resistance-trained postmenopausal women. Maturitas. 2008;59(4):394-404. doi:10.1016/j.maturitas.2008.04.002.

[93] Paolillo FR, Corazza AV, Borghi-Silva A, et al. Infrared LED irradiation applied during high-intensity treadmill training improves maximal exercise tolerance in postmenopausal women: a 6-month longitudinal study. Lasers Med Sci. 2013;28(2):415-422. doi:10.1007/s10103-012-1062-y.

[94] Paolillo FR, Borghi-Silva A, Arena R, et al. Effects of phototherapy plus physical training on metabolic profile and quality of life in postmenopausal women. J Cosmet Laser Ther. 2017;19(6):364-372. doi:10.1080/14764172.2017.1326610.

[95] Park SM, Kwak YS, Ji JG. The Effects of Combined Exercise on Health-Related Fitness, Endotoxin, and Immune Function of Postmenopausal Women with Abdominal Obesity. J Immunol Res. 2015;2015:830567. doi:10.1155/2015/830567.

[96] Pereira R, Krustrup P, Castagna C, et al. Effects of recreational team handball on bone health, postural balance and body composition in inactive postmenopausal women - A randomised controlled trial. Bone. 2021;145:115847. doi:10.1016/j.bone.2021.115847.

[97] Pereira R, Krustrup P, Castagna C, et al. Multicomponent recreational team handball training improves global health status in postmenopausal women at the long term - A randomised controlled trial. Eur J Sport Sci. 2023;23(8):1789-1799. doi:10.1080/17461391.2023.2184725.

[98] Pérez-López A, Gonzalo-Encabo P, Pérez-Köhler B, et al. Circulating myokines IL-6, IL-15 and FGF21 response to training is altered by exercise type but not by menopause in women with obesity. Eur J Sport Sci. 2022;22(9):1426-1435. doi:10.1080/17461391.2021.1939430.

[99] Phillips MD, Patrizi RM, Cheek DJ, et al. Resistance training reduces subclinical inflammation in obese, postmenopausal women. Med Sci Sports Exerc. 2012;44(11):2099-2110. doi:10.1249/MSS.0b013e3182644984.

[100] Pospieszna B, Karolkiewicz J, Tarnas J, et al. Influence of 12-week Nordic Walking training on biomarkers of endothelial function in healthy postmenopausal women. J Sports Med Phys Fitness. 2017;57(9):1178-1185. doi:10.23736/s0022-4707.16.06528-2.

[101] Rocha C, Guimaraes A, Maia B, et al. Effects of a 20-Week Concurrent Training Program on Bone Metabolism in Elderly Women. International Journal of Morphology. 2018;36(2):655-660. doi:10.4067/S0717-95022018000200655.

[102] Rashti BA, Mehrabani J, Damirchi A, et al. The influence of concurrent training intensity on serum irisin and abdominal fat in postmenopausal women. Prz Menopauzalny. 2019;18(3):166-173. doi:10.5114/pm.2019.90810.

[103] Ready AE, Naimark B, Ducas J, et al. Influence of walking volume on health benefits in women post-menopause. Med Sci Sports Exerc. 1996;28(9):1097-1105. doi:10.1097/00005768-199609000-00004.

[104] Reis JG, Costa GC, Schmidt A, et al. Do muscle strengthening exercises improve performance in the 6-minute walk test in postmenopausal women? Rev Bras Fisioter. 2012;16(3):236-240. doi:10.1590/s1413-35552012005000022.

[105] Rossi FE, Fortaleza AC, Neves LM, et al. Combined Training (Aerobic Plus Strength) Potentiates a Reduction in Body Fat but Demonstrates No Difference on the Lipid Profile in Postmenopausal Women When Compared With Aerobic Training With a Similar Training Load. J Strength Cond Res. 2016;30(1):226-234. doi:10.1519/jsc.0000000000001020.

[106] Rossi FE, Diniz TA, Neves LM, et al. The beneficial effects of aerobic and concurrent training on metabolic profile and body composition after detraining: a 1-year follow-up in postmenopausal women. Eur J Clin Nutr. 2017;71(5):638-645. doi:10.1038/ejcn.2016.263.

[107] Rossi FE, Fortaleza ACS, Neves LM, et al. Combined training (strength plus aerobic) potentiates a reduction in body fat but only functional training reduced low-density lipoprotein cholesterol in postmenopausal women with a similar training load. J Exerc Rehabil. 2017;13(3):322-329. doi:10.12965/jer.1734940.470.

[108] Rossi FE, Diniz TA, Fortaleza ACS, et al. Concurrent Training Promoted Sustained Anti-atherogenic Benefits in the Fasting Plasma Triacylglycerolemia of Postmenopausal Women at 1-Year Follow-up. J Strength Cond Res. 2018;32(12):3564-3573. doi:10.1519/jsc.0000000000001732.

[109] Saeidi A, Jabbour G, Ahmadian M, et al. Independent and Combined Effects of Antioxidant Supplementation and Circuit Resistance Training on Selected Adipokines in Postmenopausal Women. Front Physiol. 2019;10:484. doi:10.3389/fphys.2019.00484.

[110] Seo DY, Lee SR, Kim HK, et al. Independent beneficial effects of aged garlic extract intake with regular exercise on cardiovascular risk in postmenopausal women. Nutr Res Pract. 2012;6(3):226-231. doi:10.4162/nrp.2012.6.3.226.

[111] Serrano-Guzmán M, Aguilar-Ferrándiz ME, Valenza CM, et al. Effectiveness of a flamenco and sevillanas program to enhance mobility, balance, physical activity, blood pressure, body mass, and quality of life in postmenopausal women living in the community in Spain: a randomized clinical trial. Menopause. 2016;23(9):965-973. doi:10.1097/gme.0000000000000652.

[112] Shabani A, Shabani R, Dalili S, et al. The effect of concurrent endurance and resistance training on cardio-respiratory capacity and cardiovascular risk markers among sedentary overweight or obese post-menopausal women. Journal of Nursing and Midwifery Sciences. 2018;5:123. doi:10.4103/JNMS.JNMS_34_18.

[113] Shaw BS, Gouveia M, McIntyre S, et al. Anthropometric and cardiovascular responses to hypertrophic resistance training in postmenopausal women. Menopause. 2016;23(11):1176-1181. doi:10.1097/gme.0000000000000687.

[114] Shen TW, Wen HJ. Aerobic exercise affects T-wave alternans and heart rate variability in postmenopausal women. Int J Sports Med. 2013;34(12):1099-1105. doi:10.1055/s-0033-1343408.

[115] Shiotsu Y, Yanagita M. Comparisons of low-intensity versus moderate-intensity combined aerobic and resistance training on body composition, muscle strength, and functional performance in older women. Menopause. 2018;25(6):668-675. doi:10.1097/gme.0000000000001060.

[116] Sobczak K, Nowinka P, Wochna K, et al. The Effects of Nordic Walking with Poles with an Integrated Resistance Shock Absorber on Red Blood Cell Distribution and Cardiorespiratory Efficiency in Postmenopausal Women-A Randomized Controlled Trial. Biology (Basel). 2023;12(2)doi:10.3390/biology12020179.

[117] Sobrinho ACS, Benjamim CJR, Luciano de Almeida M, et al. Fourteen weeks of multicomponent training associated with flexibility training modifies postural alignment, joint range of motion and modulates blood pressure in physically inactive older women: a randomized clinical trial. Front Physiol. 2023;14:1172780. doi:10.3389/fphys.2023.1172780.

[118] Son WM, Pekas EJ, Park SY. Twelve weeks of resistance band exercise training improves age-associated hormonal decline, blood pressure, and body composition in postmenopausal women with stage 1 hypertension: a randomized clinical trial. Menopause. 2020;27(2):199-207. doi:10.1097/gme.0000000000001444.

[119] Son WH, Park HT, Jeon BH, et al. Moderate intensity walking exercises reduce the body mass index and vascular inflammatory factors in postmenopausal women with obesity: a randomized controlled trial. Sci Rep. 2023;13(1):20172. doi:10.1038/s41598-023-47403-2.

[120] Son WM, Sung KD, Cho JM, et al. Combined exercise reduces arterial stiffness, blood pressure, and blood markers for cardiovascular risk in postmenopausal women with hypertension. Menopause. 2017;24(3):262-268. doi:10.1097/gme.0000000000000765.

[121] Soori R, Rezaeian N, Khosravi N, et al. Effects of water-based endurance training, resistance training, and combined water and resistance training programs on visfatin and ICAM-1 levels in sedentary obese women. Science & Sports. 2017;32(3):144-151. doi:<https://doi.org/10.1016/j.scispo.2016.12.004>.

[122] Sugawara J, Akazawa N, Miyaki A, et al. Effect of endurance exercise training and curcumin intake on central arterial hemodynamics in postmenopausal women: pilot study. Am J Hypertens. 2012;25(6):651-656. doi:10.1038/ajh.2012.24.

[123] Taha M. Effect of High Intensity Interval Training on Endothelial Function in Postmenopausal Hypertensive Patients:Randomized Controlled Trial. International Journal of Physiotherapy. 2016;3doi:10.15621/ijphy/2016/v3i1/88908.

[124] Takahashi M, Miyashita M, Park JH, et al. Low-volume exercise training and vitamin E supplementation attenuates oxidative stress in postmenopausal women. J Nutr Sci Vitaminol (Tokyo). 2013;59(5):375-383. doi:10.3177/jnsv.59.375.

[125] Tan S, Du P, Zhao W, et al. Exercise Training at Maximal Fat Oxidation Intensity for Older Women with Type 2 Diabetes. Int J Sports Med. 2018;39(5):374-381. doi:10.1055/a-0573-1509.

[126] Tanahashi K, Akazawa N, Miyaki A, et al. Aerobic exercise training decreases plasma asymmetric dimethylarginine concentrations with increase in arterial compliance in postmenopausal women. Am J Hypertens. 2014;27(3):415-421. doi:10.1093/ajh/hpt217.

[127] Tapp LR, Signorile JF. Efficacy of WBV as a modality for inducing changes in body composition, aerobic fitness, and muscular strength: a pilot study. Clin Interv Aging. 2014;9:63-72. doi:10.2147/cia.S30048.

[128] Tayebi SM, Saeidi A, Fashi M, et al. Plasma retinol-binding protein-4 and tumor necrosis factor-α are reduced in postmenopausal women after combination of different intensities of circuit resistance training and Zataria supplementation. Sport Sci Health. 2019;15(3):551-558. doi:10.1007/s11332-019-00544-2.

[129] Trabka B, Zubrzycki IZ, Ossowski Z, et al. Effect of a MAST Exercise Program on Anthropometric Parameters, Physical Fitness, and Serum Lipid Levels in Obese Postmenopausal Women. J Hum Kinet. 2014;42:149-155. doi:10.2478/hukin-2014-0069.

[130] Ward LJ, Nilsson S, Hammar M, et al. Resistance training decreases plasma levels of adipokines in postmenopausal women. Sci Rep. 2020;10(1):19837. doi:10.1038/s41598-020-76901-w.

[131] Wen HJ, Huang TH, Li TL, et al. Effects of short-term step aerobics exercise on bone metabolism and functional fitness in postmenopausal women with low bone mass. Osteoporos Int. 2017;28(2):539-547. doi:10.1007/s00198-016-3759-4.

[132] Wieczorek-Baranowska A, Nowak A, Pilaczyńska-Szcześniak Ł. Osteocalcin and glucose metabolism in postmenopausal women subjected to aerobic training program for 8 weeks. Metabolism. 2012;61(4):542-545. doi:10.1016/j.metabol.2011.08.011.

[133] Wong A, Figueroa A. Eight weeks of stretching training reduces aortic wave reflection magnitude and blood pressure in obese postmenopausal women. J Hum Hypertens. 2014;28(4):246-250. doi:10.1038/jhh.2013.98.

[134] Wong A, Alvarez-Alvarado S, Kinsey AW, et al. Whole-Body Vibration Exercise Therapy Improves Cardiac Autonomic Function and Blood Pressure in Obese Pre- and Stage 1 Hypertensive Postmenopausal Women. J Altern Complement Med. 2016;22(12):970-976. doi:10.1089/acm.2016.0124.

[135] Wong A, Kwak YS, Scott SD, et al. The effects of swimming training on arterial function, muscular strength, and cardiorespiratory capacity in postmenopausal women with stage 2 hypertension. Menopause. 2018;26(6):653-658. doi:10.1097/gme.0000000000001288.

[136] Wong A, Figueroa A, Son WM, et al. The effects of stair climbing on arterial stiffness, blood pressure, and leg strength in postmenopausal women with stage 2 hypertension. Menopause. 2018;25(7):731-737. doi:10.1097/gme.0000000000001072.

[137] Wooten JS, Phillips MD, Mitchell JB, et al. Resistance exercise and lipoproteins in postmenopausal women. Int J Sports Med. 2011;32(1):7-13. doi:10.1055/s-0030-1268008.

[138] Wu J, Oka J, Higuchi M, et al. Cooperative effects of isoflavones and exercise on bone and lipid metabolism in postmenopausal Japanese women: a randomized placebo-controlled trial. Metabolism. 2006;55(4):423-433. doi:10.1016/j.metabol.2005.10.002.

[139] Yoon JR, Ha GC, Ko KJ, et al. Effects of exercise type on estrogen, tumor markers, immune function, antioxidant function, and physical fitness in postmenopausal obese women. J Exerc Rehabil. 2018;14(6):1032-1040. doi:10.12965/jer.1836446.223.

[140] Yoshizawa M, Maeda S, Miyaki A, et al. Additive beneficial effects of lactotripeptides and aerobic exercise on arterial compliance in postmenopausal women. Am J Physiol Heart Circ Physiol. 2009;297(5):H1899-1903. doi:10.1152/ajpheart.00433.2009.

[141] Yoshizawa M, Maeda S, Miyaki A, et al. Additive beneficial effects of lactotripeptides intake with regular exercise on endothelium-dependent dilatation in postmenopausal women. Am J Hypertens. 2010;23(4):368-372. doi:10.1038/ajh.2009.270.

[142] Zhang D, Janjgava T, Boutcher SH, et al. Cardiovascular response of postmenopausal women to 8 weeks of sprint interval training. Eur J Appl Physiol. 2019;119(4):981-989. doi:10.1007/s00421-019-04087-9.
